# Fibroblast growth factor 21 attenuates ventilator-induced lung injury by inhibiting the NLRP3/caspase-1/GSDMD pyroptotic pathway

**DOI:** 10.1186/s13054-023-04488-5

**Published:** 2023-05-22

**Authors:** Peng Ding, Rui Yang, Cheng Li, Hai-Long Fu, Guang-Li Ren, Pei Wang, Dong-Yu Zheng, Wei Chen, Li-Ye Yang, Yan-Fei Mao, Hong-Bin Yuan, Yong-Hua Li

**Affiliations:** 1 Department of Anesthesiology, Changzheng Hospital, The Second Affiliated Hospital of Naval Medical University, Shanghai, China; 2 Department of Anesthesiology, PLA No.983 Hospital, Tianjin, China; 3 Department of Pharmacology, College of Pharmacy, Naval Medical University, Shanghai, China; 4 Department of Anesthesiology and Surgical Intensive Care Unit, Xinhua Hospital Affiliated to Shanghai Jiao Tong University School of Medicine, Shanghai, China

**Keywords:** Ventilator-induced lung injury, Fibroblast growth factor 21, Pyroptosis, NLRP3, Caspase-1, Gasdermin D

## Abstract

**Background:**

Ventilator-induced lung injury (VILI) is caused by overdistension of the alveoli by the repetitive recruitment and derecruitment of alveolar units. This study aims to investigate the potential role and mechanism of fibroblast growth factor 21 (FGF21), a metabolic regulator secreted by the liver, in VILI development.

**Methods:**

Serum FGF21 concentrations were determined in patients undergoing mechanical ventilation during general anesthesia and in a mouse VILI model. Lung injury was compared between FGF21-knockout (KO) mice and wild-type (WT) mice. Recombinant FGF21 was administrated in vivo and in vitro to determine its therapeutic effect.

**Results:**

Serum FGF21 levels in patients and mice with VILI were significantly higher than in those without VILI. Additionally, the increment of serum FGF21 in anesthesia patients was positively correlated with the duration of ventilation. VILI was aggravated in FGF21-KO mice compared with WT mice. Conversely, the administration of FGF21 alleviated VILI in both mouse and cell models. FGF21 reduced Caspase-1 activity, suppressed the mRNA levels of *Nlrp3*, *Asc, Il-1β, Il-18, Hmgb1* and *Nf-κb*, and decreased the protein levels of NLRP3, ASC, IL-1β, IL-18, HMGB1 and the cleaved form of GSDMD.

**Conclusions:**

Our findings reveal that endogenous FGF21 signaling is triggered in response to VILI, which protects against VILI by inhibiting the NLRP3/Caspase-1/GSDMD pyroptosis pathway. These results suggest that boosting endogenous FGF21 or the administration of recombinant FGF21 could be promising therapeutic strategies for the treatment of VILI during anesthesia or critical care.

**Supplementary Information:**

The online version contains supplementary material available at 10.1186/s13054-023-04488-5.

## Background

Mechanical ventilation (MV) is an important component of general anesthesia and an indispensable respiratory support therapy for critically ill patients. However, MV can cause lung injury, namely ventilator-induced lung injury (VILI), which predisposes patients to inflammatory response syndrome or multiple organ failure with a mortality rate of nearly 50% [1], making it an urgent clinical problem to be solved. The primary causes of VILI are mechanical power and the duration of ventilator exposure [2]. Current studies suggest that VILI is not only a mechanical trauma but also a biotrauma, which activates a complex signaling cascade in the lung [3, 4]. Small tidal volumes and low airway pressures may reduce the morbidity and mortality of VILI [5]. In addition to canonical inflammation-related molecules including innate immune cytokines and chemokines [6], the permeability-originating obstruction response in which alveolar leakage leads to surfactant dysfunction and increases local tissue stresses also plays a critical role in VILI [7]. Thus, it is of great importance to understand the molecular mechanism of VILI and develop new preventive/therapeutic interventions.

Fibroblast growth factors (FGFs) are a family of structurally related proteins with diverse biological functions during embryonic development, tissue injury/repair, tumorigenesis, and metabolic homeostasis. To date, 23 members of the FGF family have been identified, all of which are referred to as “pluripotent” growth factors and as “promiscuous” growth factors due to their multiple actions on a wide range of cell types. FGF21, a member of the FGFs, was first identified and cloned in 2000 [8]. FGF21 is highly expressed in the liver and can be secreted into the blood [9]. Numerous clinical and basic studies have shown that FGF21 is involved in metabolic diseases such as diabetes, obesity, and nonalcoholic fatty liver disease [10–12]. Interestingly, FGF21 has been reported to be involved in lipopolysaccharide-induced lung injury [13], and the emerging roles of FGF21 in acute lung injury/acute respiratory distress syndrome, acute myocardial injury, acute kidney injury, sepsis, and other critical diseases are increasingly noteworthy [14]. Moreover, several FGF21 analogs, such as Pegbelfermin (Bristol-Myers Squibb), LY2405319 (Eli Lilly), and PF05231023 (Pfizer), have passed Phase I/II trials and were reported to be generally well tolerated and effective in treating obesity or diabetes [15–17].

Currently, there is no knowledge regarding the roles of FGF21 in VILI. We hypothesized that FGF21, which is able to protect the blood–brain barrier and reduce inflammation [18, 19], may play a role in the development and progression of VILI and, if so, further explore the underlying molecular mechanism.

## Materials and methods

### Patient enrollment and blood sample preparation

Patients undergoing general anesthesia were recruited consecutively from November 2020 to February 2021 at Shanghai Changzheng Hospital. Informed consent was obtained from all subjects, and the protocol was approved by the Ethics Committee of Biomedicine of Naval Medical University. Patients with endotracheal intubation and mechanical ventilation under general anesthesia aged 45–70 years, and ASA status I–II were enrolled. Exclusion criteria included: severe lung, liver or renal dysfunction, severe infection, malignancy, type 2 diabetes, obese patients (BMI > 30 kg/m^2^), estimated intraoperative blood loss > 500 ml, and estimated ventilation duration less than 2 h.

After the patient entered the operating room, electrocardiogram monitoring was established and radial artery catheterization was performed. Five milliliters of blood was drawn through the arterial catheter and injected into a coagulation-promoting tube, which was left at room temperature for 30 min and then centrifuged at 3000 g for 10 min. Serum was collected and stored at − 80 °C. The patients were routinely subjected to endotracheal intubation and intravenous-inhalation combined anesthesia. The ventilation parameters were set as follows: volume-controlled ventilation mode, 8–10 ml/kg tidal volume, 12 breaths/minute, 1:2 inspiration/expiration ratio, 3–5 cm H_2_O positive end-expiratory pressure, and 60–100% inhalation oxygen concentration. At the end of the operation and before extubation, 5 ml of arterial blood was extracted, and serum was collected and stored in the same way. Two tubes of serum were collected from each patient before and after mechanical ventilation. If hemolysis or lipid clots occurred in any tube of serum, the pair of samples were discarded, and the patient was excluded. Serum levels of FGF21 were measured by an ELISA kit (ab222506, Abcam, USA) according to the manufacturer’s instructions.

### Animals and the mouse VILI model

Male C57BL/6 mice were purchased from Sippr/BK Lab Animal Co., Ltd (Shanghai, China). FGF21 global knockout mice (C57BL/6N-*Fgf21*^*em1Cyagen*^, NCBI ID 56,636) were obtained from Cyagen Biosciences Inc (Santa Clara, CA, USA). Details of the breeding and identification of gene-edited mice are provided in the Additional file 1: Supplementary content. The male homozygote and wild-type mice from the same litter were used in subsequent experiments. The mice were housed in individually ventilated cages under a specific pathogen-free conditions with a controlled temperature and a 12-h light–dark cycle. All animal experiments were approved by the Ethics Committee of Biomedicine of Naval Medical University, were performed in compliance with the National Institutes of Health Guide for Care and Use of Laboratory Animals, and were reported in accordance with the Animal Research: Reporting In Vivo Experiments (ARRIVE) guidelines 2.0 [20].

The mice were fasted for 12 h before the experiment. After anesthetization by an intraperitoneal injection of ketamine (100 mg/kg) and xylazine (10 mg/kg), the mice were intubated orally with a 22-G catheter and then connected to a small animal-specific ventilator (VentElite, Harvard Apparatus, USA) and placed on a warm pad. The mice were ventilated with a tidal volume of 30 ml/kg at 70 breaths/min for 4 h [21], and control mice underwent intubation but breathed spontaneously. After the modeling, the mice were resuscitated and kept for 24 h; then, the mice were killed for subsequent experiments.

### Primary cell culture and cyclic mechanical stretch

Primary lung microvascular endothelial cells (LMEVCs) were extracted from male neonatal C57BL/6N mice (3 days old) using a tissue block attachment method (Additional file 1: Supplementary content). Complete ECM medium (ScienCell, USA) containing 5% fetal bovine serum, 1% triple antibiotics, and endothelial cell growth factors was used. Cells were identified by immunocytochemistry with the endothelial marker CD31. Third-generation LMVECs were inoculated into a 6-well Bioflex plate (Flexcell, USA). Cells were subjected to cyclic mechanical stretch (MS) in the Flexcell FX-5000 system using the following parameters: 0.5 Hz (30 times/minute); 20% max elongation; 4-h duration. After modeling, the cells were treated with rFGF21 or phosphate-buffered saline (PBS), transferred to a conventional incubator (37 °C, 5% CO_2_), and cultured for 24 h before subsequent experiments.

### Cell viability and cytotoxicity assay

Cell viability was assessed using a cell counting kit (CCK-8, Epizyme). The level of lactate dehydrogenase (LDH) in the cell culture supernatant was measured by an LDH cytotoxicity assay kit (J2380, Promega).

### FGF21 administration

Male C57BL/6 mice (6–8 weeks old, 20–24 g) were randomly divided into 5 groups (Additional file 1: Supplementary content). Recombinant mouse FGF21 (HY-P7173, MedChemExpress, Monmouth Junction, NJ) was dissolved in PBS and injected intraperitoneally at the end of VILI modeling. The dose (0.75/1.5/3.0 mg/kg) was based on previous studies of rFGF21 in the treatment of blood–brain barrier injury [19] and acute kidney injury [22].

### Bronchoalveolar lavage fluid

Bronchoalveolar lavage fluid (BALF) was collected, and the cells in BALF were stained with hematoxylin & eosin (H&E) dye for sorting and counting. The protein concentration in the supernatant was determined using a BCA protein assay kit (23,227, Thermo Fisher).

### Histology

Lung injury was assessed based on the microscopic examination of slices stained with H&E dye and a five-point numeric scores (Additional file 1: Supplementary content) [23], which was performed by a well-trained colleague in a single blind manner.

### TUNEL assay

Terminal deoxynucleotidyl transferase-mediated dUTP nick-end labeling (TUNEL) was performed to evaluate cell death using a commercial kit (G1501, Servicebio) according to the manufacturer’s instructions.

### Evans blue index

The mice were injected with 0.1 ml of 0.5% Evans blue dye (sc-203736A, Santa Cruz) through the femoral vein. Equal weights of tissue were taken from each of the two lungs; one tissue block was measured to determine its dry weight, and the other was examined for its dye content (Additional file 1: Supplementary content). The Evans blue index is expressed as the amount of Evans blue dye per unit weight of lung tissue (ng/mg tissue).

### Oxidation stress measurement

Reactive oxygen species (ROS) were detected by a dichlorodihydrofluorescein diacetate (DCFH-DA) probe with a BioTek Gen 5 instrument (Ex 488 nm, Em 525 nm) and a fluorescence microscope (Leica DMI4000B, Germany). Myeloperoxidase (MPO) activity was measured using a commercial kit (A044-1-1, Jiancheng Biotech) according to the manufacturer’s instructions. The total antioxidant capacity in lung tissues/cell homogenate was measured using a commercial kit (S0119, Beyotime). Superoxide dismutase (SOD) activity was measured using a kit (S0109, Beyotime) based on the nitroblue tetrazolium reduction reaction.

### Mitochondrial membrane potential and apoptosis detection

This experiment was performed using a trichrome fluorescent staining kit (C1071, Beyotime) according to the manufacturer’s instructions. Mitochondria were labeled with Mito-tracker Red CMXRos (red), dead cells were labeled with Annexin V-FITC (green), and nuclei were labeled with Hoechst 33,342 (blue). The fluorescence intensity was measured by a BioTex Gen 5 instrument (Biotex, USA), and Hoechst 33,342 was used as an internal reference to compare the differences in the fluorescence intensities of mitochondrial and dead cells between the groups.

### Caspase-1 activity

Caspase-1 activity was assessed using a commercial kit (C1101, Beyotime) according to the manufacturer's instructions. Briefly, lung tissues were homogenized and lysed. The lysates were incubated with Ac-YVAD-*p*NA (2 mM) at 37 °C for 2 h. Then, the absorbance was measured at 405 nm using a BioTek Gen 5 instrument (BioTek, USA), and the activity was calculated according to the standard curve.

### Quantitative real-time PCR

Lung tissues were homogenized in RNAiso reagent (9108, Takara), and total RNA was extracted and reverse-transcribed into cDNA using PrimeScript RT Master Mix (RR036A, Takara). Primers (Additional file 1: Supplementary Table 3) were designed using Primer Express software (Applied Biosystem, USA). The reaction was performed in a QuantStudio 5 system (Thermo Fisher, USA) with a QuantiNova SYBR Green PCR Kit (208,056, Qiagen). The housekeeping gene *Actb* was used as an internal control, and the relative gene expression was analyzed using the 2^−ΔΔCt^ method.

### Western blotting

Western blotting was performed as described previously [24]. The tissues were homogenized in RIPA lysis buffer (P0013B, Beyotime) containing a protease inhibitor (GRF101, Epizyme). The samples were separated on a 10% SDS–PAGE gel, and the proteins were transferred onto a nitrocellulose membrane, which was blocked in protein-free rapid blocking buffer (PS108, Epizyme). The membranes were incubated with the primary antibody (Additional file 1: Supplementary Table 4) overnight at 4 °C, washed and incubated with IRDye-conjugated secondary antibodies (LI-COR, Lincoln, NE) for 1 h at room temperature. Images were obtained using an Odyssey infrared imaging system (LI-COR). Quantitative analysis was performed using ImageJ software (National Institutes of Health, USA).

### Statistics

Data normality was assessed by the Shapiro–Wilk test. The data are presented as mean ± standard error of mean (mean ± SEM) or median [quartile 1, quartile 3] according to the distribution. The intergroup difference was analyzed by Student’s *t*-test, paired *t*-test, or one-way ANOVA followed by LSD post hoc test according to the grouping design (Prism 9.0, GraphPad software, CA). *P* < 0.05 was considered statistically significant.

## Results

### FGF21 is induced after MV in patients and mice

**Fig. 1:**
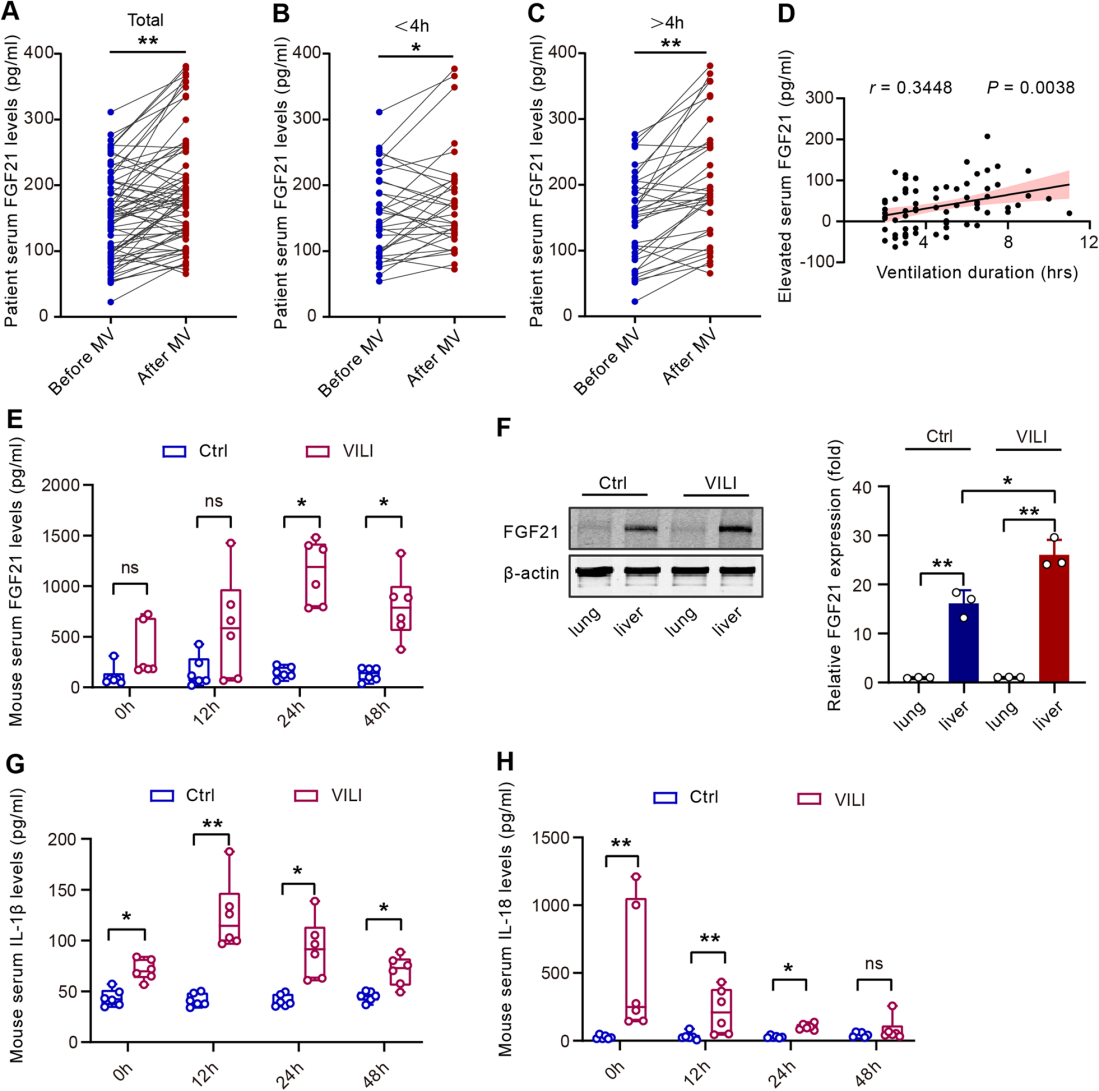
FGF21 is induced after MV in patients and mice.** A.** The average serum FGF21 increased in patients after mechanical ventilation (*N* = 69); **B.** The average serum FGF21 increased in patients after short duration (< 4 h) of mechanical ventilation (*N* = 33); **C.** The average serum FGF21 increased in patients after long duration (> 4 h) of mechanical ventilation (*N* = 36); **D.** The elevated FGF21 level was positively correlated with duration of mechanical ventilation; **E.** Serum FGF21 increased in VILI mice (*N* = 6, 0 h referred to the time point immediately after VILI modeling); **F.** FGF21 protein expression in mice lung/liver tissues after mechanical ventilation; **G.** Serum IL-1β increased in VILI mice (*N* = 6); **H.** Serum IL-18 increased in VILI mice (*N* = 6); ns, no significance, ^*^P < 0.05, ^**^P < 0.01; MV, mechanical ventilation; VILI, ventilator-induced lung injury

We collected blood samples from 69 patients who underwent MV during surgery and compared the baseline and postsurgery serum levels of FGF21 (patient characteristics in Table 1). The serum levels of FGF21 in these patients were significantly induced by MV (190.6 ± 10.2 vs. 152.9 ± 7.9 pg/ml, Fig. 1A). When these patients were divided into two subgroups based on the duration of ventilation (< 4 h and > 4 h), we found a more pronounced increase in the latter group: the mean serum FGF21 level increased from 154.9 ± 11.4 pg/ml to 175.5 ± 13.3 pg/ml in patients with MV < 4 h (Fig. 1B), while it increased from 151.1 ± 11.4 pg/ml to 204.4 ± 15.0 pg/ml in patients with MV > 4 h (Fig. 1C). Additionally, Pearson correlation analysis showed that the elevated serum FGF21 levels were positively correlated with the duration of MV (Fig. 1D).

**Table 1:** Patient characteristics

	Total (*N* = 69)
Age (yr)	57 ± 1
Female (%)	11 (16)
Body mass index (kg/m^2^)	23.7 ± 0.4
ASA physical status (%)	
I	21 (30)
II	48 (70)
Mechanical ventilation duration (hr)	4.5 [3.0, 6.5]
Surgery type (%)	
Spine surgery	48 (70)
Brain surgery	11 (16)
Gastrointestinal surgery	10 (14)
Baseline FGF21 (pg/ml)	152.9 ± 7.9
Postoperative FGF21 (pg/ml)	190.6 ± 10.2

Categorical variables are presented as N (%), continuous variables are presented as mean ± SEM or median [quartile 1, quartile 3]. ASA, American Society of Anesthesiologists; FGF21, fibroblast growth factor 21

Next, we investigated the influence of MV on serum and tissue FGF21 levels in a mouse VILI model. Serum FGF21 levels in VILI mice at 24 h or 48 h after MV were significantly higher than those in the control mice (Fig. 1E). We also examined FGF21 protein expression in the liver and lung tissue of mice with VILI and found that FGF21 protein expression was barely detectable in the lung but was expressed abundantly in the liver. Moreover, hepatic FGF21 protein levels were upregulated by VILI (Fig. 1F). IL-1β and IL-18, two proinflammatory cytokines, were induced by MV (Fig. 1G&H). These results suggest that the increased serum FGF21 may be associated with VILI.

### Deletion of FGF21 aggravates VILI in mice

**Fig. 2:**
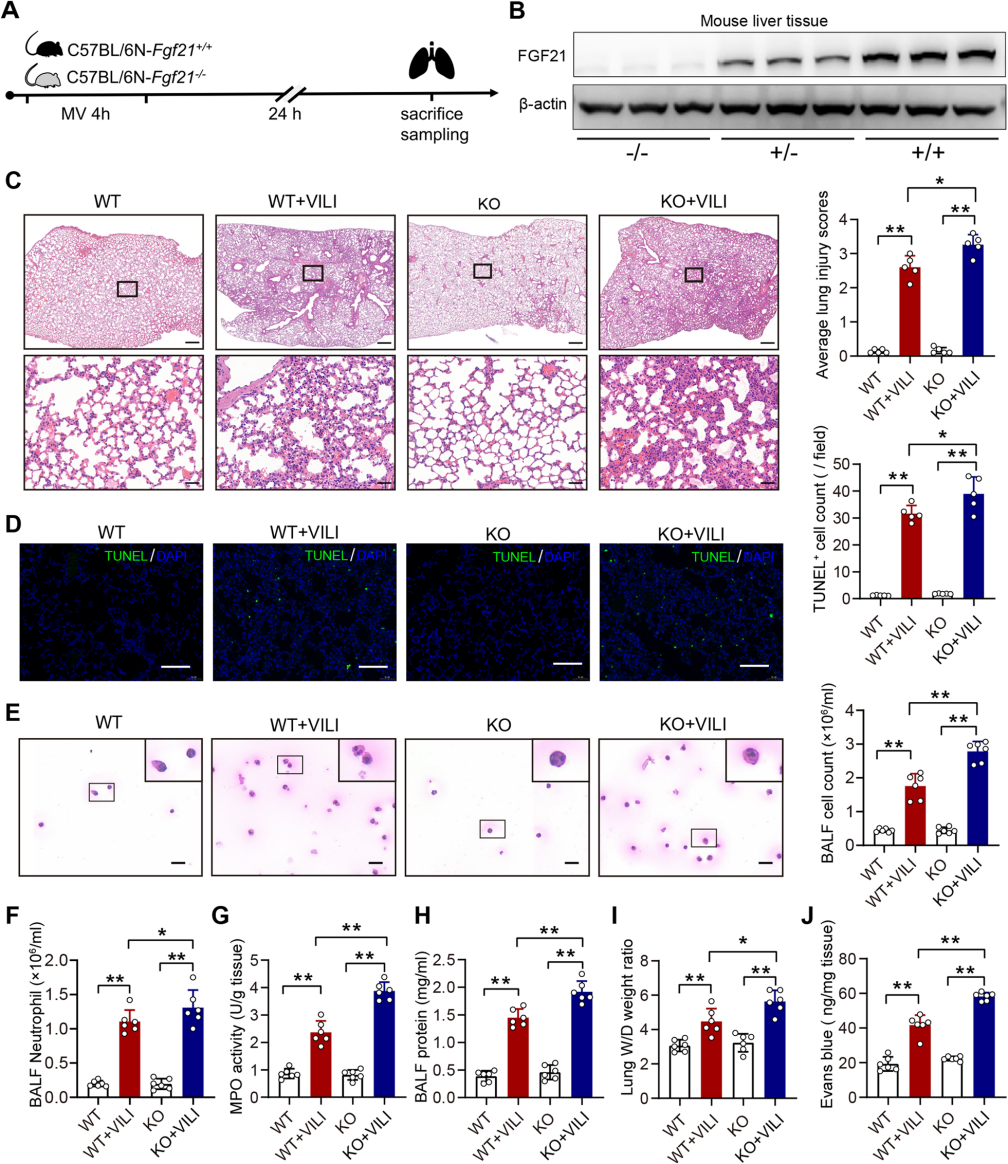
Deletion of FGF21 aggravates VILI in mice.** A.** Timeline of the loss-of-function experiment; **B.** FGF21 protein expression in liver of KO/WT mice (*N* = 3); **C.** Hematoxylin–eosin staining of lung tissues, and the average lung injury scores analysis of lung slices (*N* = 5, scale bar = 500 μm in low-power images and = 50 μm in amplified images); **D.** TUNEL staining of lung tissues, and dead cell count of lung slices (*N* = 5, scale bar = 100 μm); **E.** H&E staining of exfoliated cells in BALF, and the total cell count in BALF (*N* = 6, scale bar = 20 μm); **F.** Neutrophil count in BALF; **G.** MPO activity in lung tissue (*N* = 6); **H.** Protein concentration in BALF (*N* = 6); **I.** Wet/dry ratio of lung tissue (*N* = 6); **J.** The content of Evans blue dye in lung tissue (*N* = 6); ns, no significance, ^*^P < 0.05, ^**^P < 0.01; WT, wild-type; KO, knockout; MV, mechanical ventilation; VILI, ventilator-induced lung injury; TUNEL, TdT-mediated dUTP nick end labeling; DAPI, 4',6-diamidino-2-phenylindole; BALF, bronchoalveolar lavage fluid; MPO, Myeloperoxidase

We next used a mouse strain with global knockout of FGF21 to examine the pathophysiological role of FGF21 in VILI (Fig. 2A). Using immunoblotting, we confirmed the deficiency of FGF21 in KO mice (Fig. 2B). H&E staining showed that there were no abnormalities in the unventilated lung tissues of KO mice and WT mice. However, VILI pathologies, including pulmonary edema and inflammatory cell infiltration, were evident in WT mice and more pronounced in KO mice (Fig. 2C). TUNEL staining showed that the number of TUNEL^+^ cells was increased by VILI in WT mice and, to a great extent, in KO mice (Fig. 2D). We used H&E staining to assess the number of cells in BALF and found that the number of total cells in the BALF of KO mice was significantly higher than that in WT mice in the VILI model (Fig. 2E), most of which were neutrophils (Fig. 2F). We also measured MPO activity, which is a marker of neutrophils, and found that FGF21 knockout further promoted MPO activity in the context of VILI (Fig. 2G). BALF protein levels were induced by VILI and were more pronounced in KO mice (Fig. 2H), suggesting that FGF21 gene knockout aggravated pulmonary microvascular barrier disruption during VILI. The results of the wet/dry ratio of lung tissue and Evans blue staining demonstrated that vascular barrier permeability was damaged by VILI in WT mice and, to a great extent, in FGF21-KO mice (Fig. 2I&J). These aggravated VILI-related pulmonary pathologies in FGF21-KO mice indicate that endogenous FGF21 may be a protective factor in VILI.

### FGF21 treatment alleviates VILI in mice

**Fig. 3:**
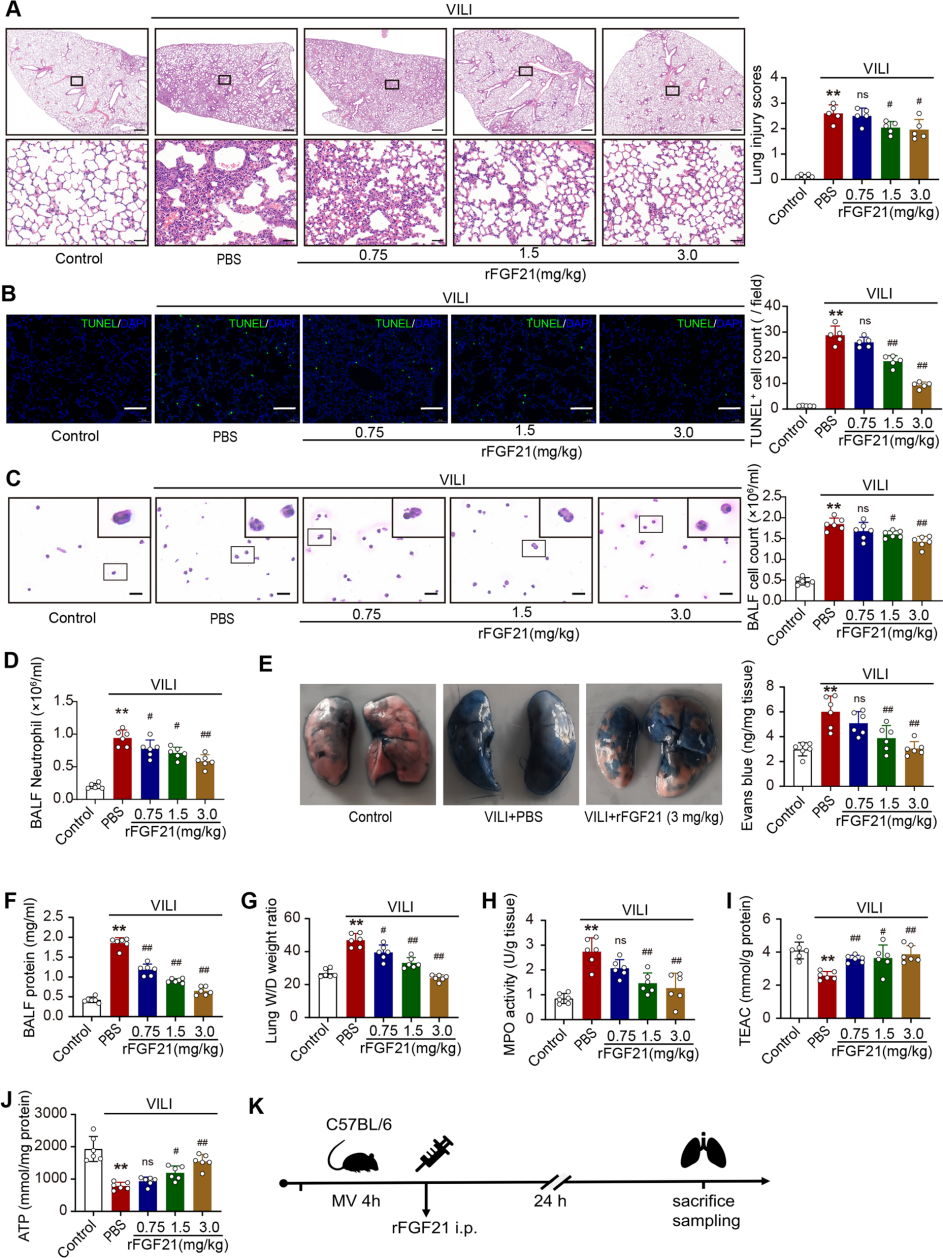
FGF21 treatment alleviates VILI in mice. **A.** Hematoxylin–eosin staining of lung tissues and average lung injury scores analysis of lung slices (*N* = 5, scale bar = 500 μm in low-power images and = 50 μm in amplified images); **B.** TUNEL staining of lung tissues and dead cells counting of lung slices (*N* = 5, scale bar = 100 μm); **C.** H&E staining of exfoliated cells and total cell counting in BALF (*N* = 6, scale bar = 20 μm); **D.** Neutrophil count in BALF; **E.** The content of Evans blue dye in lung tissue; **F.** Protein concentration in BALF; **G.** Wet/dry ratio of lung tissue; **H.** MPO activity in lung tissue. **I.** Trolox-equivalent antioxidant capacity in mice lung tissue. **J.** ATP level in mice lung tissue; **K.** Timeline of the treating experiment. ns, no significance, ^*^P < 0.05, ^**^P < 0.01 vs. Control; ^#^*P* < 0.05, ^##^*P* < 0.01 vs. PBS; MV, mechanical ventilation; TUNEL, TdT-mediated dUTP nick end labeling; DAPI, 4',6-diamidino-2-phenylindole; BALF, bronchoalveolar lavage fluid; MPO, Myeloperoxidase; TEAC, Trolox-equivalent antioxidant capacity

To further validate the therapeutic effect of FGF21, we administered mouse-origin recombinant FGF21 (rFGF21) in a mouse VILI model. H&E staining showed that ventilation-induced damages, including massive inflammatory cell infiltration, severe interstitial edema, intra-alveolar hemorrhage, were found alleviated in groups with mid- and high doses of rFGF21. In contrast, a low dose of rFGF21 failed to protect against VILI (Fig. 3A). Similar dose-dependent therapeutic effects were observed by TUNEL staining (Fig. 3B). H&E staining showed that the number of total cells as well as neutrophils in BALF was decreased significantly after rFGF21 administration, suggesting that FGF21 may play a role in reducing the infiltration and exudation of proinflammatory cells in the lung (Fig. 3C and D). Evans blue accumulation was enhanced in VILI mice and reduced by rFGF21 (Fig. 3E). Accordingly, the protein content in BALF increased significantly after VILI but decreased significantly in all FGF21-treated groups (Fig. 3F). The increased W/D ratio of the lung was also reduced in FGF21-treated groups (Fig. 3G). These results suggest that FGF21 alleviates microvascular barrier damage and pulmonary edema in a dose-dependent manner. We next determined oxidative stress in lung tissues. MPO activity induced by VILI was significantly decreased by rFGF21 treatment (Fig. 3H). The decrease in total antioxidant capacity, which was evaluated by Trolox-equivalent antioxidant capacity (TEAC), was reversed by rFGF21 treatment (Fig. 3I). Moreover, rFGF21 treatment restored the ATP levels in the mitochondrial fraction of lung tissue from VILI mice (Fig. 3J). These results suggest that FGF21 might be able to alleviate VILI in mice.

### FGF21 treatment reduces endothelial injury and downregulates pulmonary interstitial pro-fibrosis factors

**Fig. 4:**
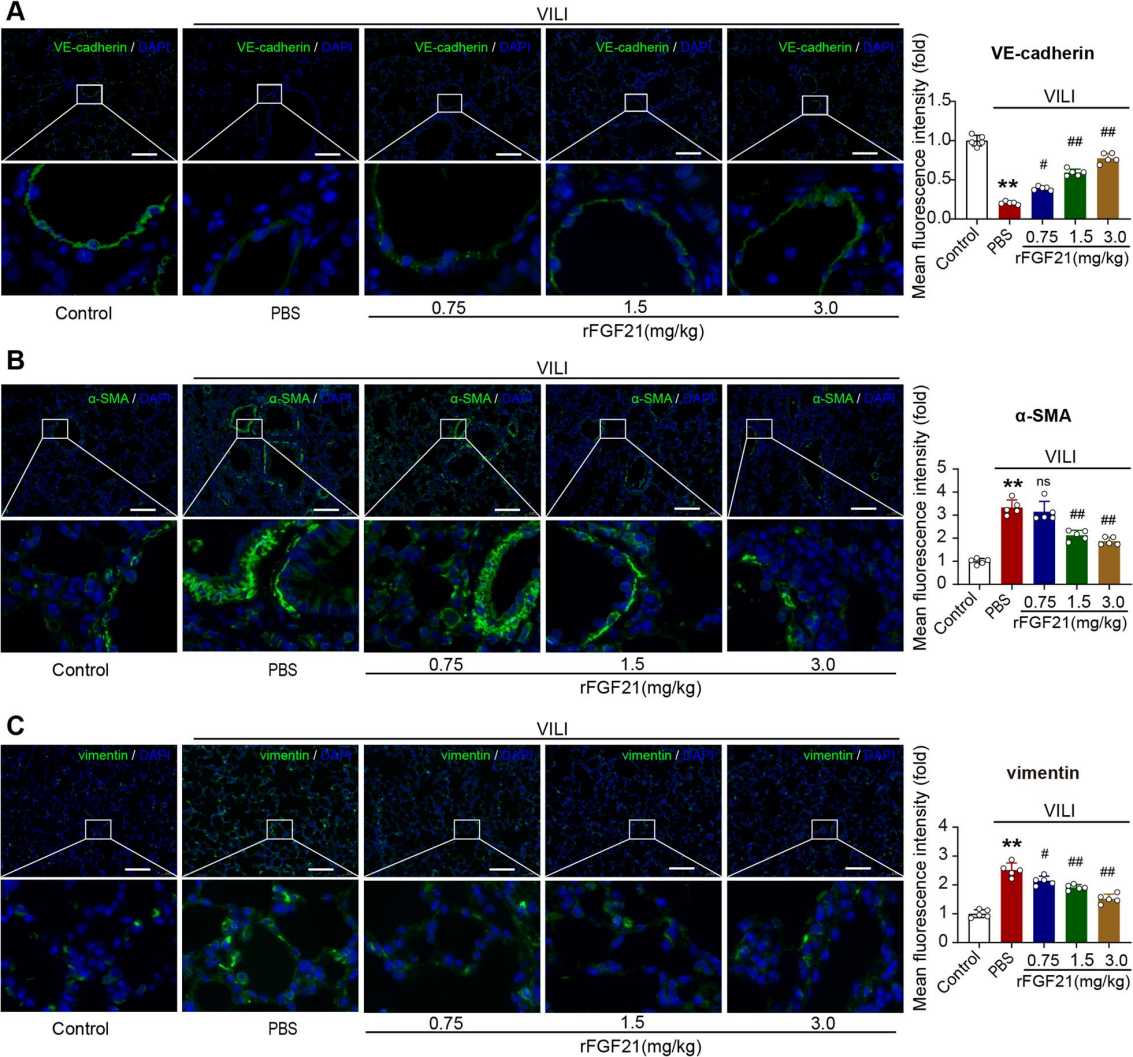
FGF21 treatment inhibits endothelial injury and downregulates pulmonary interstitial pro-fibrosis factors.** A.** Immunofluorescence of vascular endothelial marker, VE-cadherin, in lung tissue; **B.** Immunofluorescence of pulmonary interstitial marker, α-SMA, in lung tissue; **C.** Immunofluorescence of pulmonary interstitial marker, vimentin, in lung tissue; *N* = 6 in A-C; ns, no significance, ^*^P < 0.05, ^**^P < 0.01 vs. Control; ^#^*P* < 0.05, ^##^*P* < 0.01 vs. PBS; Bar scale = 100 μm; MV, mechanical ventilation; DAPI, 4',6-diamidino-2-phenylindole

Next, we evaluated endothelial injury and pulmonary interstitial changes in a VILI mouse model. In situ immunofluorescence staining of VE-cadherin, a marker of endothelial junctions, clearly showed that the VE-cadherin fluorescence intensity in the microvessels of intact blood-perfused lungs was significantly decreased after MV. However, FGF21 treatment attenuated the decline of VE-cadherin fluorescence intensity in a dose-dependent manner (Fig. 4A). We also detected pulmonary interstitial fibrosis tendency by immunofluorescence staining of pro-fibrosis factors α-SMA and vimentin. The result showed that α-SMA was induced in lung tissues by MV, and this effect was partially prevented by mid- and high doses of rFGF21 (Fig. 4B). Immunofluorescence staining of vimentin showed that the three doses of rFGF21 efficiently suppressed MV-induced vimentin expression in lung tissue (Fig. 4C). These results indicate that FGF21 treatment protects against MV-induced endothelial injury and inhibits pro-fibrosis factors.

### FGF21 treatment ameliorates MS-induced injury in a cell model

**Fig. 5:**
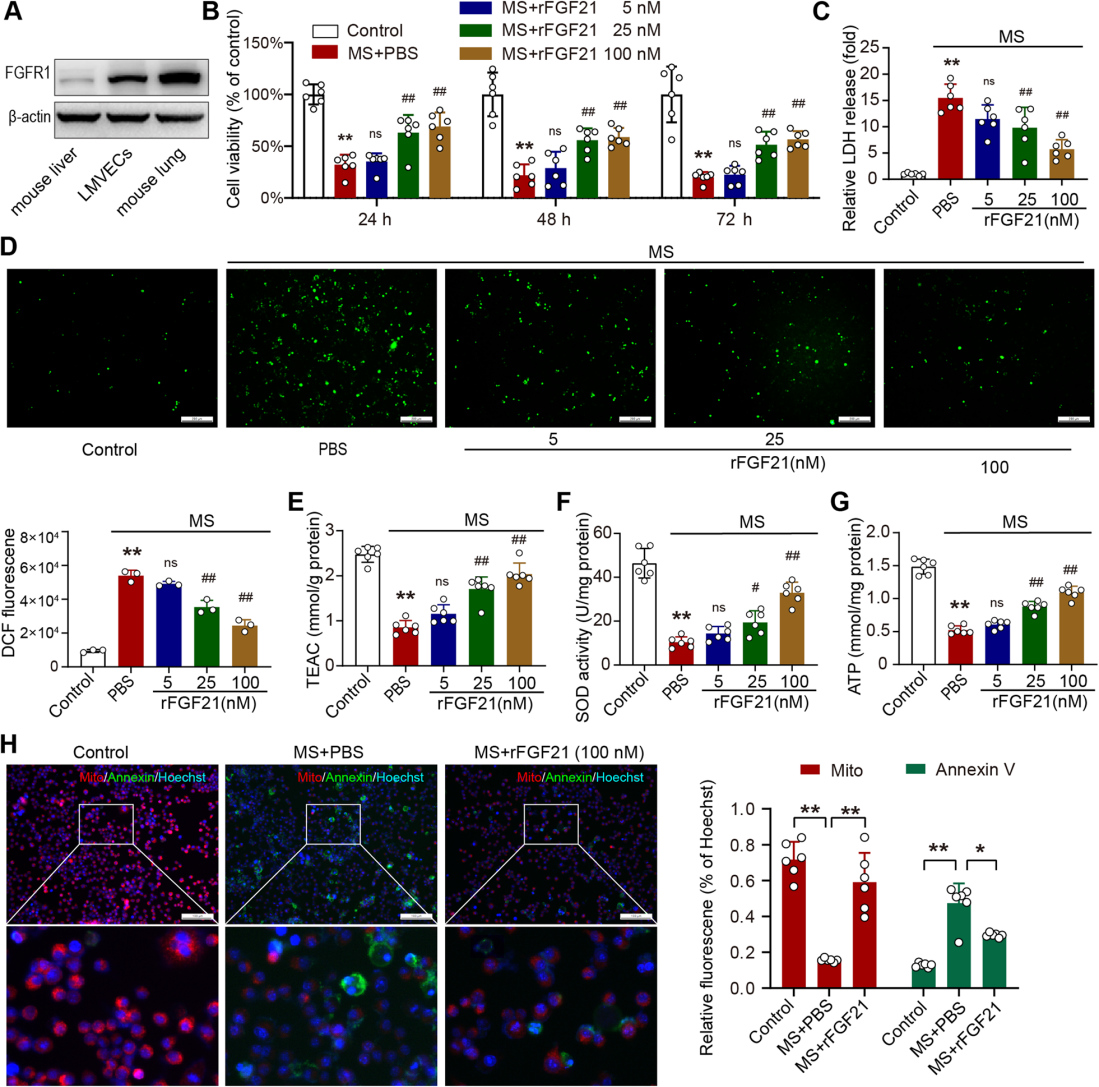
FGF21 treatment ameliorates MS-induced injury in a cell model. **A.** FGFR1 expression in mouse liver, lung, and primary lung microvascular endothelial cells;** B.** Cell viability assessment by CCK8 assay (*N* = 6); **C.** Relative LDH level in cell culture supernatant (*N* = 6); **D.** Detection of reactive oxygen species by dichlorodihydrofluorescein probe in situ (*N* = 3, scale bar = 100 μm); **E.** Trolox-equivalent antioxidant capacity in cells (*N* = 6); **F.** Superoxide dismutase activity in cells (*N* = 6). **G.** Adenosine triphosphate level in cells (*N* = 6); **H.** Mitochondrial membrane potential and cell death staining (*N* = 6, scale bar = 100 μm); ns, no significance, ^*^P < 0.05, ^**^P < 0.01 vs. Control; ^#^*P* < 0.05, ^##^*P* < 0.01 vs. PBS; FGFR1, fibroblast growth factor receptor 1; LMVESs, lung microvascular endothelial cells; MS, mechanical stretch; LDH, lactate dehydrogenase; DCF, dichlorodihydrofluorescein; TEAC, Trolox-equivalent antioxidant capacity; SOD, Superoxide dismutase

FGF21 can impact whole-body metabolism and biological functions through multiple mechanisms, including endocrine, paracrine, and autocrine effects [25]. To clarify how FGF21 protects against VLLI in cellular level, we established a mechanical stretch model in cultured primary LMVECs, in which FGFR1, the major receptor of FGF21, was highly expressed (Fig. 5A). Cell viability was decreased significantly by cyclic MS stress and was partially restored by FGF21 treatment (Fig. 5B). The levels of LDH in the culture medium were examined to determine cell integrity. We observed that the LDH content in the culture medium was increased significantly by MS, whereas rFGF21 treatment reduced this change (Fig. 5C). We also evaluated oxidative stress in this cell model. Intracellular ROS levels were determined by a DCFH-DA probe. Cyclic MS stress significantly induced ROS levels in LMVECs, while rFGF21 treatment inhibited ROS production (Fig. 5D). The TEAC assay results showed that FGF21 rescued the impaired antioxidant capacity induced by MS stress in LMEVCs (Fig. 5E). The activity of SOD, an important antioxidant enzyme, was decreased significantly by MS and was restored by FGF21 treatment (Fig. 5F). Mitochondrial dysfunction has been reported to play a key role in the pathophysiology of VILI [26]. We also measured the influence of rFGF21 on mitochondrial dysfunction in the cell model. Intracellular ATP levels were dramatically reduced by MS stress, but were rescued by rFGF21 (Fig. 5G). Mitochondrial membrane potential was examined by immunofluorescent staining. The cells underwent MS stress exhibited an obvious increase in Annexin immunofluorescence (green) and a decrease in mitochondrion immunofluorescence (red), which was largely inhibited by rFGF21 (Fig. 5H). These results indicate that FGF21 ameliorates cellular injury, oxidative stress, and mitochondrial dysfunction in a cell model of mechanical stretch.

### FGF21 protects against VILI by inhibiting the NLRP3/Caspase-1/GSDMD pyroptotic pathway

**Fig. 6:**
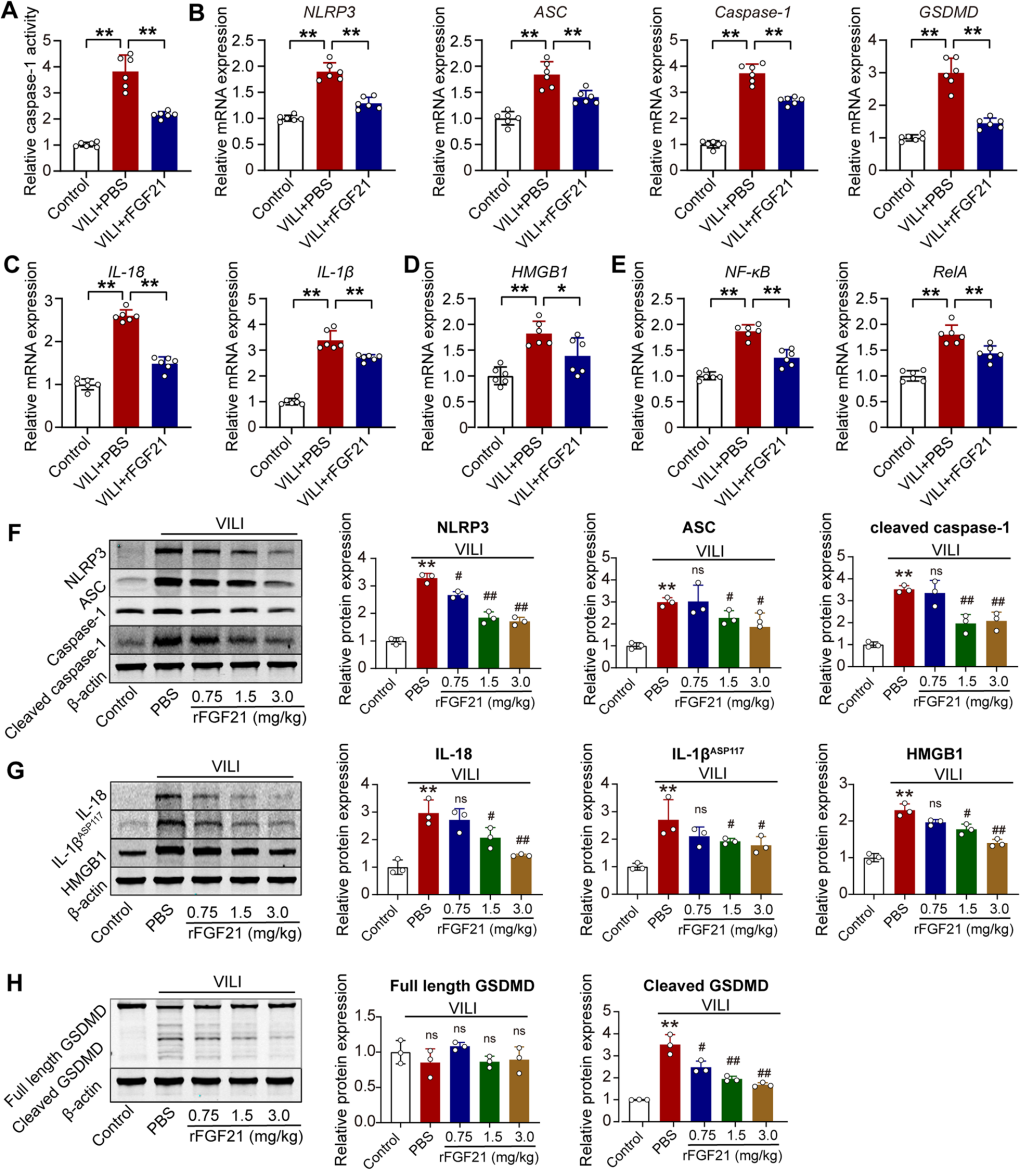
FGF21 protects against VILI via inhibiting NLRP3/Caspase-1/GSDMD pyroptotic pathway. **A.** Relative caspase-1 activity in mice lung tissue; **B-E.** Relative mRNA expression levels of key nodes in NLRP3/caspase-1/GSDMD pathway (*N* = 6, ^*^P < 0.05, ^**^P < 0.01); **F–H.** Relative protein expression levels of key nodes in NLRP3/caspase-1/GSDMD pathway (*N* = 3; ns, no significance, ^*^P < 0.05, ^**^P < 0.01 vs. Control; ^#^*P* < 0.05, ^##^*P* < 0.01 vs. PBS). Lung tissue samples in Fig. 6 are from the same batch of mice in Fig. 3

Next, we sought to decipher the molecular mechanism underlying the protective effect of FGF21 on VILI. Pyroptosis, which is a form of programmed cell death, is critically involved in acute lung injury and VILI [27, 28]. Previously, pyroptosis was believed to be mediated by the NLRP3 inflammasome [29]; currently, pore formation in the cell membrane mediated by gasdermin (GSDM) proteins, especially caspase-1 cleaved GSDMD [30], is thought to be the crucial characteristic of pyroptosis. We examined the activity of caspase-1 in the lung tissues and found that caspase-1 activity was triggered (~ fourfold) in VILI model mice, while the administration of rFGF21 abolished this change (Fig. 6A). The mRNA levels of pyroptotic factors, including *Nlrp3, Asc, Casp1* and *Gsdmd*, increased significantly in VILI mouse’s lung tissue (Fig. 6B). Accordingly, the mRNA levels of *Il-1β* and *Il-18*, two proinflammatory factors released by pyroptotic cells, were enhanced by VILI but suppressed by rFGF21 treatment (Fig. 6C). Similar changes in *high mobility group protein 1* (*Hmgb1*), a delivery protein of pyroptosis [31], were observed (Fig. 6D). Gene expression of the proinflammatory factors *Nf-κb* and *Rela* was also induced by VILI and inhibited by rFGF21 (Fig. 6E).

We further examined the influence of FGF21 on the protein expression of pyroptotic factors. Similar to the changes in mRNA levels, the protein levels of NLRP3 and ASC were significantly upregulated in VILI mice, and this effect was prevented by FGF21 treatment (Fig. 6F). Total caspase-1 was slightly induced by VILI, while cleaved caspase-1, the activated form of caspase-1, was markedly induced by VILI, and FGF21 treatment successfully suppressed this change (Fig. 6F). Mature IL-18 and IL-1β^Asp117^ were significantly induced in the lung tissue of VILI mice and were attenuated by rFGF21 treatment in a dose-dependent manner (Fig. 6G). A similar change in HMGB1 was observed (Fig. 6G). Finally, we examined the cleavage of GSDMD. Obvious cleavage of GSDMD was noted in the lung tissue of VILI mice, which was partially blocked by rFGF21 in a dose-dependent manner (Fig. 6H). These results suggest that the protective effect of FGF21 might be associated with the inhibition of the NLRP3/Caspase-1/GSDMD pyroptotic pathway.

## Discussion

The pathogenesis of VILI is multifactorial and complex, resulting predominantly from interactions between ventilator-related factors and patient-related factors. In the present study, we provided evidence that circulating levels of FGF21 were increased in both patients and mice with longtime mechanical ventilation. Using a mouse strain with FGF21 deficiency, we demonstrated that VILI pathologies were further aggravated by FGF21 deletion, suggesting that FGF21 may be an endogenous mechanism in response to VILI stimuli. Moreover, we showed that the administration of FGF21 successfully ameliorated VILI in a mouse model and rescued mechanical stretch-induced injury in a cell model. Mechanistically, we found that inhibiting the NLRP3/Caspase-1/GSDMD pyroptotic pathway may contribute to the protective effect of FGF21 against VILI.

The first interesting finding is that FGF21 is induced after VILI, and we speculated that the elevated circulating FGF21 might be mainly synthesized and secreted by the liver. Previous studies have shown significantly elevated levels of FGF21 in patients with type 2 diabetes, nonalcoholic fatty liver and obesity [32, 33]. In addition, circulating FGF21 was increased in response to cardiac stress [34], ischemic stroke [35], limb ischemia/reperfusion injury [36], and toxic kidney injury [37]. We found that serum FGF21 tended to increase and peaked at approximately 24 h after VILI modeling in mice, which was reported for the first time. It is reported in some public databases that FGF21 is not expressed in normal lung tissues or lung cells (Additional file 1: Fig. S4), and we also found that FGF21 was enriched in the lung after systemic administration of recombinant FGF21 (Additional file 1: Fig. S4). These findings supported the hypothesis that exogenous rFGF21 was enriched in the lung after rFGF21 medication. Nevertheless, we cannot exclude that endogenous FGF21 might be triggered after mechanical ventilation. As this preliminary conclusion is based on only one single time point after medication, further studies on pharmacology and pharmacokinetic profiles of rFGF21 may be needed in the future.

We also report for the first time that VILI is more severe in FGF21-knockout mice, suggesting that endogenous FGF21 may be a protective factor. In addition to results from the loss-of-function of FGF21, we further explored the therapeutic effect of recombinant FGF21 and the results were supportive. As some FGF21-related candidate drugs, such as FGF21 analogs (Pegbelfermin, LY2405319, PF05231023) have been clinically tested to treat diabetes and other metabolic disorders, our findings strongly suggest that testing the efficacy of these FGF21 analogs in patients at high risk of VILI may be necessary.

The FGF receptor family includes FGFR1, FGFR2, FGFR3, FGFR4, and an FGFR-like protein-FGFR5 [38], among which FGFR1 is abundantly expressed in fibroblasts, smooth muscle cells, respiratory ciliated cells, and endothelial cells in the lung (Additional file 1: Fig. S5). We knockdown FGFR1 by small interfering RNA and found that the protective effect of FGF21 was attenuated, indicating that FGFR1 plays a key role in mediating the biological function of FGF21 (Additional file 1: Fig. S6). These findings may help to explain the molecular mechanisms underlying the protective role of FGF21 in the lung, and further study on the specific ligand–receptor interaction and signal transduction process is still needed.

Oxidative stress is an important link in the pathophysiological development of lung injury. During MV, alveolar epithelial cells and vascular endothelial cells produce large amounts of ROS in response to cyclic stretch and shear forces [39]. Mitochondria are the most important sites of ROS production. Under oxidative stress, ROS overload leads to mitochondrial dysfunction, decreased ATP synthesis capacity, reduced mitochondrial membrane potential, and reduced scavenging free radical capacity, leading to a further increase in ROS and forming a vicious cycle [40]. When there is an imbalance between high levels of ROS and antioxidant capacity, cells are unable to maintain normal redox homeostasis, leading to cellular damage and inflammatory responses [41]. Kang et al. [42] found that FGF21 could reduce neuroinflammation and oxidative stress by regulating the NF-κB pathway and the AMPK/AKT pathway in an aged diabetic mouse model. Zhang et al. [43] found that FGF21 had a therapeutic effect on pulmonary fibrosis by activating the Nrf-2 pathway and thus inhibiting oxidative stress and extracellular matrix deposition. Our findings are in line with these results and indicate that FGF21 inhibits the increase in ROS production, restores the antioxidant capacity of cells, and stabilizes the membrane potential and function of mitochondria.

Additionally, we found an association between FGF21 and pyroptosis. Pyroptosis, which is a form of lytic cell death, plays a vital role in innate immune; however, aberrant pyroptosis can contribute to injury in multiple organs. The activation of caspase-1 triggers pyroptosis, and GSDMD leads to pore formation, resulting in the cleavage of inflammatory cytokines [44]. Since caspase-1 plays a central role in inducing pyroptosis and the NLRP3/caspase-1 axis has been well studied, it is possible to reduce pyroptosis by regulating NLRP3. Wei et al*.* [45] reported that FGF21 improved intimal hyperplasia in diabetic mice, which was associated with inhibition of the FGFR1/Syk/NLRP3 pathway. Chen et al*.* [46] showed that FGF21 inhibited pyroptosis in human umbilical vein endothelial cells by suppressing ROS production. In our study, we found that the induced mRNA levels of NLRP3 inflammasome, the protein expression of IL-18 and IL-1β, and the cleavage of GSDMD were all inhibited by FGF21 treatment. Therefore, our results indicate a critical role of the NLRP3/Caspase-1/GSDMD pyroptotic pathway in the pathophysiology of VILI, which is consistent with two recently published works [47, 48], and further point out a potential therapeutic effect of FGF21 against VILI-related pyroptosis. However, the regulatory mechanisms of FGF21 on pyroptosis, especially in VILI, may need further investigation.

As mentioned above, the liver is a major manufacturer of FGF21, and we hypothesized that elevated circulating FGF21 is secreted by the liver. However, how the liver responds to volutrauma/biotrauma in the lung and whether this is a causal or correlation relationship is poorly understood. It may be the effect of specific cytokines or mediators released by the lung or through neurohumoral regulation. We believe that screening signaling molecules relating to the cross talk between the lung and the liver after mechanical ventilation using systemic methods (transcriptomics, proteomics, and metabolomics) might be a promising approach to answer this conundrum.

There are several limitations in this study. First, only male mice were used in this study for concerns about confounding contributions from the hormone cycle in female mice since our research target is also a circulating hormone. In addition, some studies reported the sex difference in metabolic responses and pharmacological effects of FGF21 [49, 50], while gender differences in lung injury are rarely reported. Whether FGF21 benefits VILI both in male and in female is an intriguing question meriting further investigation. Second, the experimental design lacked a group of mice ventilated with normal tidal volume. The tidal volume of 30 ml/kg is classic in mouse model while exceeds what would be used in any clinical setting. It will be helpful in the clinical translation of the findings if regular ventilated mice were tested in future study.

## Conclusion

Our study indicates that the increase in serum FGF21 levels after MV might be an endogenous protective response. Treatment with rFGF21 protects against VILI in vivo and in vitro by inhibiting the NLRP3/Caspase-1/GSDMD pathway. These findings suggest that FGF21 might be a promising pharmacological tool in the battle against VILI.

## Supplementary Information

**Additional file 1.** Detailed methods and supplementary results.

## Data Availability

The datasets generated and/or analyzed during the current study are available from the corresponding authors on reasonable request.

## Abbreviations

FGF21: Fibroblast growth factor 21
VILI: Ventilator-induced lung injury
NLRP3: NOD-, LRR- and pyrin domain-containing 3
KO: Knockout
WT: Wild-type
ASC: Apoptosis-associated speck-like protein containing a CARD
IL: Interleukin
HMGB1: High mobility group box 1
GSDMD: Gasdermin D
MV: Mechanical ventilation
ASA: American Society of Anesthesiologists
BMI: Body mass index
ELISA: Enzyme-linked immunosorbent assay
LMEVCs: Lung microvascular endothelial cells
MS: Mechanical stretch
PBS: Phosphate-buffered saline
LDH: Lactate dehydrogenase
BALF: Bronchoalveolar lavage fluid
BCA: Bicinchoninic acid
H&E: Hematoxylin and eosin
TUNEL: Terminal deoxynucleotidyl transferase-mediated dUTP nick-end labeling
ROS: Reactive oxygen species
DCFH-DA: Dichlorodihydrofluorescein diacetate
MPO: Myeloperoxidase
SOD: Superoxide dismutase
PCR: Polymerase chain reaction
SDS–PAGE: Sodium dodecyl sulfate polyacrylamide gel electrophoresis
SEM: Standard error of mean
TEAC: Trolox-equivalent antioxidant capacity
