# Supplementary material for: Fibroblast growth factor 21 attenuates ventilator-induced lung injury by inhibiting the NLRP3/caspase-1/GSDMD pyroptotic pathway
Source: Crit Care. 2023 May 22;27:196. doi: 10.1186/s13054-023-04488-5 (PMC10204208; doi:10.1186/s13054-023-04488-5)
Supplement: Supplementary file 1 — Additional file 1. Detailed methods and supplementary results. [file 13054_2023_4488_MOESM1_ESM.docx]

**Fibroblast growth factor 21 attenuates ventilator-induced lung injury by inhibiting NLRP3/Caspase-1/GSDMD pyroptotic pathway**

Peng Ding^1,2,#^, Rui Yang^1,#^, Cheng Li^1,#^, Hai-Long Fu^1^, Guang-Li Ren^2^, Pei Wang^3^, Dong-Yu Zheng^1^, Wei Chen^1^, Li-Ye Yang^1^, Yan-Fei Mao^4,*^, Hong-Bin Yuan^1,*^, and Yong-Hua Li^1,*^

^1^Department of Anesthesiology, Changzheng Hospital, the Second Affiliated Hospital of Naval Medical University, Shanghai, China;

^2^Department of Anesthesiology, PLA No.983 Hospital, Tianjin, China;

^3^Department of Pharmacology, College of Pharmacy, Naval Medical University, Shanghai, China;

^4^Department of Anesthesiology and Surgical Intensive Care Unit, Xinhua Hospital Affiliated to Shanghai Jiao Tong University School of Medicine, Shanghai, China.

^#^Contributed equally

**Corresponding author:**

Prof. Yong-Hua Li, [liyonghua1207@smmu.edu.cn](mailto:liyonghua1207@smmu.edu.cn)

Prof. Hong-Bin Yuan, [jfjczyy@aliyun.com](mailto:jfjczyy@aliyun.com)

Prof. Yan-Fei Mao, [maoyanfei@xinhuamed.com.cn](mailto:maoyanfei@xinhuamed.com.cn)

**Methods and materials**

1. **FGF21 knockout mouse**
   1. **Gene editing and breeding**

In this experiment, two male and two female C57BL/6N-Fgf21^em1cyagen^ mice, each 8 weeks old, were provided by Cyagen Biosciences Inc. The target gene is mouse *Fgf21* (NCBI code 56636) and it is globally knocked out. The target gRNA sequence is: forward 5'-TGTGTCAAATATCACGCGTCAGG-3', reverse 5'-GAGTGGGTAACCACGATTGTTGG-3'. The knockout region spans the three exons of the *Fgf21* gene (**Supplementary Figure 1A**).

Healthy 8-week-old wild-type C57BL/6N mice were purchased from the Experimental Animal Center of Naval Military Medical University. They were bred with the gene-edited mice at a 1:2 ratio of male/female to obtain F1-generation heterozygous mice. After the F1 generation mice were sexually mature (about 8 weeks old), the heterozygous mice were self-bred to obtain the F2 generation mice with the following genotypes: 50% heterozygote, 25% homozygote, and 25% wild type **(Supplementary Figure 1B)**. The male homozygote and wild-type mice from the same litter were used for subsequent experiments, and the heterozygous mice were used for expansion breeding.

- 1. **Identification**

When the mice were 3 weeks old, a small section of the tail was cut and the total DNA was extracted using a genotyping assay kit (D7283, Beyotime). The sequence of the primers used in PCR amplification was presented in Supplemental Table 1. Electrophoresis (150V, 30 min) was performed using 1.5% agarose gel. The gel was then exposed and the photograph was taken using the Syngene imager system (Synoptics, UK). Homozygous mice had only a 906 bp band, wild-type mice had only a 624 bp band, and heterozygous mice had both 906/624 bp bands **(Supplementary Figure 1C)**.

**Supplementary Table 1.** Primer sequence in genotyping

| F1 | 5’-CAGACCCAGGAGTGTAGACTTCAG-3’ |
| --- | --- |
| R1 | 5’-CCAGTGGTTCCATTCTCAGTAC-3’ |
| R2 | 5’-AGCTGAGAAGACACTAAGGCTGTC-3’ |

1. **Mouse VILI model**
   1. Transoral tracheal intubation

Ketamine was injected intraperitoneally, and after obtaining satisfactory anesthesia, the upper incisors of the mice were suspended on a homemade device for intubation in mice (**Supplementary Figure 2A),** which has been granted a Chinese utility model patent (**Supplementary Figure 2B**, Patent No. ZL 2021-2-1550170.9). A chalazion forceps was extended into the mouth to expose the pharynx, and a rice-sized cotton ball was taken with ophthalmic forceps and dabbed to remove oral secretions. A cold light source lamp was applied to the thorax of mice, and the laryngeal cavity was observed. A light spot with a diameter of about 1~2 mm was seen, which opened and closed in line with the respiratory rate, and this was the mouse glottis (Yellow arrow, **Supplementary Figure 2C**). A 20G catheter was inserted under direct vision and the end of the catheter was connected to the outer barrel of a 1ml syringe. A drop of water was injected in to seal the barrel and the water droplet was seen to swing back and forth with breathing (with an amplitude of at least 2 cm), which meant that the catheter was right in the trachea. If the water droplet does not move or flutters slightly, that means the catheter is in the esophagus and needs to be reintubated.

- 1. Grouping and treatment

Male C57BL/6 mice (6-8 weeks old, weighed 20-24 g) were randomly divided into 5 groups (N=6 per group): Control (intubation + spontaneous breath + PBS 0.2 ml), PBS (intubation + MV 4h + PBS 0.2 ml), Low (intubation + MV 4h + rFGF21 0.75 mg/kg), Mid (intubation + MV 4h + rFGF21 1.5 mg/kg), High (intubation + MV 4h + rFGF21 3.0 mg/kg). There have been few reports on the use of rFGF2 in lung injury, so we set the doses in the present study based on previous researches of rFGF21 in the treatment of blood-brain barrier injury (PMID: 29648978) and acute kidney injury (PMID: 32210821).

- 1. Mouse VILI model
     1. Set ventilator parameters: tidal volume 30 ml/kg, respiratory rate 70 breaths/min, inspiration: expiration ratio 1:2.
     2. The mice were placed in the supine position, fixed on a heating plate with tapes, connected to the ventilator, and started ventilation, and the duration time was set to 4 hours.
     3. Observe the thoracic undulation of the mouse, it should be symmetrical on both sides, if it is not symmetrical, it may be that the catheter is inserted too deep to cause single-lung ventilation, then the catheter should be withdrawn appropriately until the respiratory movement are symmetrical.
     4. When the mice showed a body movement or rapid shaking of whiskers during ventilation (usually occurred at 2 hours after the initiation of ventilation), additional anesthetic should be added to prevent accidental extubation.
     5. Regarding the mice in the control group, they were intubated, fixed on the heating pad, and kept breathing spontaneously. The respiratory status of the mice was checked every 30 minutes using the water-sealed barrel to prevent the mice from asphyxiation due to the obstruction of the catheter by secretions.
     6. After ventilation, disconnect the tube, and observe for a moment, the mice resume autonomous breathing, connect the water-sealed barrel, and observe the fluctuation of water droplets, if it is greater than 2 cm, then the tube can be withdrawn.
     7. After extubation, put the mice back in the prone position, use the heat pad and heat lamp to prevent hypothermia, and resume feeding and water intake after the mice are fully awakened and can move freely.

1. **HE staining and histopathologic scoring**

Mouse lung tissue was fixed in 4% paraformaldehyde and made into paraffin sections with a 5 μm thickness. The slide was immersed in xylene to dissolve all the wax away. After thorough de-waxing, the slide was passed through several changes of alcohol to remove the xylene, then thoroughly rinsed in water. The slide was stained with hematoxylin and then “blued” by treatment with an alkaline solution. Remove the excess staining solution and apply the Eosin counterstain. Following the eosin stain, the slide was passed through several changes of alcohol to remove all traces of water, then rinsed in several baths of xylene to render it completely transparent. A thin layer of polystyrene mountant was applied, followed by a glass coverslip.

Ten fields of view (200×) per slide were randomly selected for observation and analysis, with indicators including interstitial lung edema, inflammatory cell infiltration. A five-point pathology score was assigned to the lung injury condition, where a score of 0 indicates normal lung tissue or very mild pathological changes, 1 indicates mild, limited pathological changes, 2 indicates moderate pathological changes, 3 indicates severe, extensive pathological changes, and 4 indicates very severe pathological changes. Scoring was done by a well-trained colleague who was blind to the grouping.

1. **TUNEL assay**

The slides were immersed in antigen repair solution, boiled for 15 minutes, cooled naturally to room temperature, and rinsed 3 times with PBS buffer for 5 minutes each. Proteinase K solution (20 ug/ml) was added to cover the tissue area and incubated at 37°C for 20 minutes. Then the equilibration buffer was added and incubated for 10 min at room temperature. The TdT incubation buffer (containing recombinant TdT enzyme, FITC-12-dUTP labeling solution, and equilibration buffer) was then added and incubated for 1 hour at 37°C in a light-proof wet box, followed by rinsing the sections with PBS buffer, 4 times × 5 min. The slides were immersed in DAPI solution and incubated for 8 min at room temperature, protected from light. The sections were washed with PBS buffer 3 times × 5 min, dabbed to remove excess liquid, and sealed with an antifade mounting medium. Microscopic examination was performed as soon as possible using a fluorescent microscope. Five non-overlapping fields of view were randomly selected for each slice and counted by an observer who was unaware of the grouping.

1. **Bronchoalveolar lavage fluid assay**
   1. BALF collect

Mice were anesthetized and intubated, and 1 ml of saline was injected through the endotracheal tube. The alveoli were repeatedly lavaged three times, then the lavage fluid was collected (0.7 ml minimum).

- 1. Cell counting

BALF was centrifugated at 1000 g for 10 minutes. The cell pellets were resuspended in red blood cell lysis buffer (Servicebio) and centrifugated again. The supernatant was removed and the cell pellet was resuspended in PBS and made into single-cell suspension. Cell suspension and Trypan blue dye (Sangon, China) were mixed in an equal volume and added to the Conutstar plate (Inno-Alliance Biotech Inc., Wilmington, USA) to detect the total number of cells.

- 1. Exfoliated cells staining

BALF was centrifugated at 1000 g for 10 minutes. The cell pellets were resuspended in methanol and fixed for 15 minutes at room temperature. After centrifugation at 1000 g for 5 minutes, the supernatant was removed and the cell pellet was resuspended in PBS. The cell suspension was added onto a glass slide and dried (not in an oven). The slides were fixed in 95% alcohol for 20 minutes, and then were washed by PBS for 2 times. The slides were immersed in hematoxylin dye solution for 2 minutes, and rinsed with tap water. Then the status of nuclear staining was confirmed under a microscope. The slides were then immersed in eosin dye solution for 1 minute, rinsed with tap water, dried, sealed with neutral resin and observed under a microscope. The neutrophils can be identified by the multi-lobulated nuclei, and macrophages can be identified by the single oval nuclei.

- 1. Protein concentration measurement

BALF was centrifugated at 1000 g for 10 minutes, and the supernatant was collected and placed on ice. Bovine serum albumin was diluted in PBS and made into standards of different concentrations. The samples and standards were added to a 96-well plate, followed by the working solution (bicinchoninic acid and copper sulfate in a 50:1 ratio). The plate was incubated at 37°C for 30 minutes and the absorbance at 562 nm was read by a microplate reader (BioTek, USA). The protein concentration in samples was calculated according the standard curve.

1. **Wet/dry weight ratio**

After the mouse was anesthetized, the chest was opened, the lung tissue was removed, the right lower lobe was separated, and the blood on the surface was blotted dry with a clean paper. The weight of the lobe was weighed and recorded as wet weight (W). The lobe was then dried at 80 °C for 48 hours until the weight did not change and the current weight was weighed and recorded as dry weight (D). A W/D weight ratio was then calculated accordingly.

1. **Evans blue index**

After anesthesia, the mice were injected with 0.1 ml of 0.5% Evans blue dye (sc-203736A, SantaCruz) through femoral vein. The mice were observed for 30 minutes and were seen to turn blue at the tail and corners of the mouth. The left auricle of the mouse was cut open and iced PBS was perfused through the right ventricle until the effluent from the left auricle was clarified and free of blue color. Both lungs were excised, rinsed once with PBS, and blotted dry with clean paper. Weigh 50 mg of left lung, reduce into small pieces, add 100 μl of iced PBS as homogenization medium, and homogenize in a tissue grinder (70 Hz, 2 min). Add 400 μl formamide solution to the homogenate and incubate in an oven at 37°C for 24 hours. The homogenate was centrifuged (5000 g, 10 min) and the supernatant was collected. Weigh 50 mg of right lung tissue, place in an oven at 60°C, dry for 48 hours until the weight no longer changes, and weigh the dry weight of the lung tissue. On the other hand, Evans blue dye was diluted with formamide solution, configured into different concentrations of standards, and incubated with the sample for 24 hours. The absorbance at 630 nm was read by a microplate reader (Biotech, USA), and the content of Evans blue dye in the samples was calculated according to the standard curve. The Evans blue index is expressed as the amount of Evans blue dye per unit weight of lung tissue (ng/mg tissue).

1. **Myeloperoxidase activity**

Myeloperoxidase (MPO) activity was measured using a commercial kit (A044-1-1, Jiancheng Biotech) according to the manufacturer’s instructions. The decomposition of 1 μmol hydrogen peroxide in 1 gram tissue in the reaction system at 37°C was defined as one unit of myeloperoxidase activity.

1. **Trolox-equivalent antioxidant capacity**

The cells were scraped off the petri dish (no trypsin needed), resuspended with ice PBS, and lysed with an ultrasonic comminuter. The supernatant was collected after the homogenate was centrifuged at 4°C 12000g for 5 minutes. Tissue preparation is similar to the cell. The samples, blank control, and standard samples (Trolox) were added to a 96-well plate, followed by the working solution (S0119, Beyotime). After incubation for 5 minutes at room temperature, the absorbance at 734 nm was read by the BioTek Gen 5 microplate reader. The total antioxidant capacity of the sample was calculated according to the standard curve and presented as Trolox-Equivalent Antioxidant Capacity (TEAC).

1. **ATP measurement**

Cells/tissues were lysed and homogenized on ice using the lysis buffer provided in the ATP assay kit (S0026, Beyotime). The homogenate was centrifugated at 12000 g for 5 minutes, then the supernatant was collected and the protein concentration was measured. The working solution (containing fluorescein and firefly luciferase) was added to an all-black 96-well plate, and the test samples were added 5 minutes later and mixed well. The RLU value was read by a luminometer (BioTek, USA). ATP content of the sample was calculated according to the standard curve and divided by the protein concentration of the corresponding sample to convert to nmol/mg protein.

1. **Immunofluorescence**

Paraffin section preparation, dewaxing, hydration, antigen repair, and other steps are described above. The sections were immersed in QuickBlock (P0260, Beyotime) solution and blocked at room temperature for 60 min. Primary antibody (**Supplementary Table 2**) was added to the section to ensure that the tissue was all covered. The section was placed in a wet box sheltered from light and incubated overnight at 4°C. The slices were removed the next day and rinsed 3 times with PBS buffer for 5 minutes each time. Secondary antibody solution (**Supplementary Table 2**) was added and the sections were incubated at room temperature for 1 hour. Wash the slices and then DAPI staining solution (C1005 Beyotime) was added. After 5 minutes of incubation and being washed 3 times, the slices were sealed with antifade mounting medium (P0126, Beyotime) and transferred to the darkroom as soon as possible for fluorescence microscopy, image collection and analysis.

**Supplementary Table 2.** Antibodies used in immunofluorescence

| Antibody | Dilution | Number | Manufacturer |
| --- | --- | --- | --- |
| α-SMA | 1:100 | GB111364 | Servicebio |
| VE-cadherin | 1:100 | GB14013 | Servicebio |
| vimentin | 1:100 | GB12192 | Servicebio |
| Donkey anti-rabbit secondary antibody, Alexa Flour 488 | 1:1000 | A21206 | Thermo Fisher |

1. **Cell culture and mechanical stretch**
   1. Primary lung microvascular endothelial cells (LMVECs) culture

Male newborn C57BL/6N mice (3 days old) were used in this experiment. The mice were disinfected with 75% alcohol, and be careful not to soak the head to prevent alcohol inhalation. Then the mice were moved into a clean bench and quickly decapitated with sharp scissors. The right ventricle of mice was perfused with cold PBS, then the lung lobes was removed, cut into small pieces (1 mm), and coated in the culture dish, which was pre-coated with ECM medium (ScienCell, USA). The Petri dish was turned over and cultured for 2 h at 37°C with 5% CO_2_. After that, the dish was taken out and 2 ml of ECM medium (containing 5% fetal bovine serum, 1% penicillin + streptomycin + amphotericin B triple antibiotics, and endothelial cell growth factor) was added slowly along the side wall. The dish was incubated for 48 hours without changing the medium. Microscopic observation shows the cell migration around the tissue block, gently remove the tissue block with a pipette tip, change all the medium and continue the culture, and change the medium every 48 hours thereafter. The first passage (in a 1:1 ratio) was performed when the cell confluence was about 60-80%. Accutase (Merck Millipore, USA) was used to digest cells to avoid damage. The second passage (in a 1:2 ratio) was performed when the cells were about 80% confluent, and thereafter.

- 1. Cell identification by immunocytochemistry

Immunocytochemistry was performed when cells grow to 3^rd^ generation. Cells were fixed using paraformaldehyde, blocked for 1 hour at room temperature using quick block solution (P0260, Beyotime), and incubated in anti-CD31 antibody (1:100, AF6408, Beyotime) overnight at 4°C. Cells were washed for three times with PBS and then incubated in FITC-labeled secondary antibody (1:500, A21206, Thermo Fisher) for 1 hour at room temperature. After staining nuclei with DAPI, observe the cells with a fluorescence microscope as soon as possible.

The image of immunocytochemistry (see below) showed that more than 95% of the primary cultured cells were stained positive with CD31, that is, the vast majority of cells were lung microvascular endothelial cells.

**
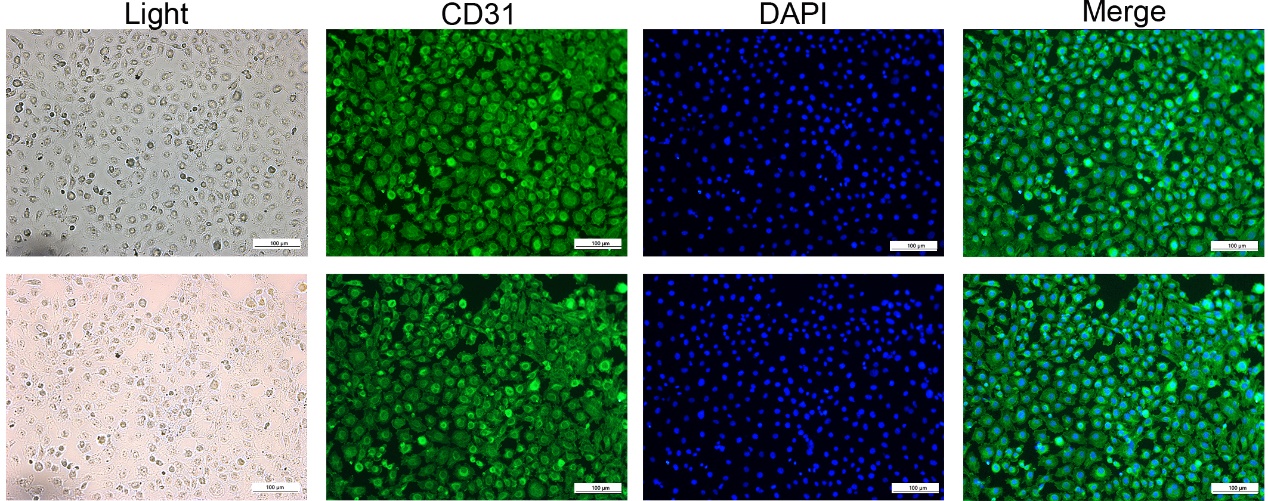
**

- 1. Mechanical stretch

The day before the experiment, the 3^rd^ generation of LMVECs was digested and prepared into a single-cell suspension. The cells were counted and inoculated into a 6-well Bioflex plate (Flexcell, USA), with 1×10^6^ cells and 2 ml ECM medium per well. The plate was incubated with 5 % CO_2_ at 37°C for 24 hours. The cells were divided into 5 groups with 3 wells in each group: Control group (sham treatment + PBS 2 ul), PBS group (MS+PBS 2 ul), Low (MS + rFGF21 200 ng/5 nM), Mid (MS + rFGF21 1 ug/25 nM), High (MS + rFGF21 2 ug/100 nM). The next day, the cells were subjected to mechanical stretch in an incubator attached to the Flexcell FX-5000 system using the following parameters: sine wave, frequency 0.5 Hz (30 times/minutes), max elongation 20%, duration 4 hours. The sham-treated cells were also cultured in the same incubator but without MS. After modeling, cells were treated with rFGF21 or PBS, transferred to a conventional incubator (37°C, 5% CO_2_), and continued to be cultured for 24 hours for follow-up experiments.

1. **Transfection of small interfere RNA**

The small interfering RNA (siRNA) targeting FGFR1 (NM_010206.3) was designed using Invitrogen BLOCK-iT™ RNAi Designer (https://rnaidesigner.thermofisher.com/rnaiexpress/) and synthesized by Thermo Fisher. Lung microvascular endothelial cells were transfected with FGFR1 siRNA or control siRNA (Silencer^®^ Negative Control #5 siRNA, Thermo Fisher) using the Xfect RNA Transfection Reagent (631450, Takara).

1. **Cell viability**

Cell viability was assessed using the cell counting kit-8 (CCK-8, Epizyme). The cells were rinsed once with PBS, and 2 ml of fresh medium (containing 200 μl of CCK-8 reagent) was added. After incubation at 37°C for 2 hours, the absorbance (A) at 450 nm was determined by a microplate reader (BioTek, USA). Other cells in the same batch of the experiment and under the same culture conditions were measured again 48 hours and 72 hours after modeling. The cell viability was calculated as follows: cell viability=(A_experiment_-A_blank_)/(A_control_-A_blank_)×100%, in which the experimental wells refer to cells undertaken MS and rFGF21 treatment + medium + CCK-8, the control wells were cells undertaken MS and vehicle treatment + medium + CCK-8, and the blank wells were cell-free medium + CCK-8.

1. **Lactate dehydrogenase measurement**

The LDH cytotoxicity assay kit (J2380) was purchased from Promega. The cells were seeded into a six-well plate and subjected to mechanical stretch, followed by rFGF21 treatment or vehicle treatment. The LDH release reagent was added to a “maximum enzyme activity (max)” well and then fully mixed. The plate was incubated for one hour and centrifugated at 400 g for 5 minutes. The supernatant was collected and then incubated at 25°C for 30 minutes in dark with INT (2-p-iodophenyl-3-nitrophenyl tetrazolium chloride), diaphorase, and lactate. The absorbance (A) at 490 nm was read and the cytotoxicity was calculated as (A_treat_-A_control_)/(A_max_-A_control_)×100%. Relative LDH release was compared by normalization of the control group.

1. **ROS detection**

The ROS probe DCFH-DA (S0033, Beyotime) was diluted in serum-free medium at 1:1000 and loaded in situ on the cells, which were incubated at 37°C for 30 minutes, and then were washed with serum-free medium for three times. The fluorescence intensity of DCF was read by BioTek Gen 5 instrument (Ex 488 nm, Em 525 nm) and then the plate was observed using an inverted fluorescence microscope (Leica DMI4000B, Germany).

1. **SOD activity**

SOD activity was measured using a kit based on nitroblue tetrazolium reduction reaction. Lung tissues or cells were homogenized with lysis buffer provided in the kit. The supernatant was collected after centrifugation at 12000 g for 5 minutes, and protein concentration was measured by the BCA method. Samples were incubated with WST-8, Xanthine Oxidase, and the reaction start-up solution at 37°C for 30 minutes, and the absorbance (A) at 450 nm was read by a microplate reader (BioTek, USA). Refer to the following formula to calculate the inhibition rate: Inhibition rate = (A_Blank 1_-A_sample_)/(A_Blank 1_-A_Blank 2_)×100%, in which Blank 1 refers to the reaction system without samples and Blank 2 refers to the reaction system without start-up solution. When the inhibition rate is 50% in the above xanthine oxidase coupling reaction system, the SOD activity in the reaction system is defined as one unit, thus, the SOD activity in the sample can be calculated as inhibition rate/(1-inhibition rate) units accordingly.

1. **Mitochondrial membrane potential and apoptosis detection**

This experiment was performed using a fluorescence staining kit (C1071, Beyotime) according to the manufacturer’s instructions. Cell cultures were aspirated and cells were washed with PBS. Adherent cells were digested with trypsin (EDTA free), centrifuged at 1000 g for 5 minutes, collected, and resuspended in PBS, together with cells collected from wash waste before. After centrifugation, 5×10^4^ cells were resuspended with Annexin-FITC binding buffer (188 μl), Mito-Tracker Red CMXRos (2 μl), Annexin V-FITC (5 μl), and Hoechst 33342 dye (5 μl) was added, gently mixed, and incubated in dark for 30 minutes at room temperature. Centrifuged again for 5 minutes, collected cells, resuspended with 100 μl Annexin-FITC binding buffer, made a cell smear, and immediately observed under a fluorescence microscope. Mitochondria were labeled with red fluorescence, dead cells with green fluorescence, and nuclei with blue fluorescence. In another experiment, the stained cells were added to an all-black 96-well plate, and the fluorescence intensity was read with BioTex Gen 5 instrument (Biotex, USA) using the following parameters: Mito-Tracker Red CMXRos (Ex/Em: 579/599 nm), Annexin V-FITC (Ex/Em: 492/520 nm), and Hoechst 33342 (Ex/Em: 350/461 nm). Hoechst 33342 was used as an internal reference to compare the differences in fluorescence of mitochondrial and dead cells between the groups.

1. **Caspase-1 activity**

Caspase-1 activity was assessed using a commercial kit (C1101, Beyotime) according to the manufacturer's instructions. Briefly, lung tissues were homogenized and lysed on ice. The supernatants of the homogenates were collected after centrifugation at 20000 g, and the protein concentration was determined using a Bradford protein assay kit (P0006, Beyotime). The lysates were incubated with Ac-YVAD-*p*NA (2 mM) at 37°C for 2 hours. After incubation, the absorbance was read at 405 nm using BioTek Gen 5 instrument (BioTek, USA) and the activity was calculated from a standard curve. Fold changes in caspase-1 activity were determined by comparison with control samples.

1. **Quantitative real-time PCR**

Lung tissues were homogenized in RNAiso reagent (9108, Takara) and total RNA was extracted and reverse transcripted into cDNA using PrimeScript RT Master Mix (RR036A, Takara). Primers (**Supplementary Table 3**) were designed using the Primer Express software (Applied Biosystem, USA) and synthesized by Sangon biotech company (Shanghai, China). The reaction was performed in QuantStudio 5 system (Thermo Fisher, USA) with QuantiNova SYBR Green PCR Kit (208056, Qiagen). The housekeeping gene *Actb* was used as internal control, and the relative gene expression was analyzed using the 2^−ΔΔCt^ method.

**Supplementary Table 3.** Primer sequence

| Gene | NCBI ID | Sequence |
| --- | --- | --- |
| *Nlrp3* | 216799 | F: 5’-ATTACCCGCCCGAGAAAGG-3’ |
|  |  | R: 5’-TCGCAGCAAAGATCCACACAG-3’ |
| *Asc* | 66824 | F: 5’-CTTGTCAGGGGATGAACTCAAAA-3’ |
|  |  | R: 5’-GCCATACGACTCCAGATAGTAGC-3’ |
| *Casp1* | 12362 | F: 5’-ACAAGGCACGGGACCTATG-3’ |
|  |  | R: 5’-TCCCAGTCAGTCCTGGAAATG-3’ |
| *Il-18* | 16173 | F: 5’-GTGAACCCCAGACCAGACTG-3’ |
|  |  | R: 5’-CCTGGAACACGTTTCTGAAAGA-3’ |
| *Il-1b* | 16176 | F: 5’-GCAACTGTTCCTGAACTCAACT-3’ |
|  |  | R: 5’-ATCTTTTGGGGTCCGTCAACT-3’ |
| *Gsdmd* | 69146 | F: 5’-ATGCCATCGGCCTTTGAGAAA-3’ |
|  |  | R: 5’-AGGCTGTCCACCGGAATGA-3’ |
| *Hmgb1* | 15289 | F: 5’-GGCGAGCATCCTGGCTTATC-3’ |
|  |  | R: 5’-GGCTGCTTGTCATCTGCTG-3’ |
| *Nfkb1* | 18033 | F: 5’-ATGGCAGACGATGATCCCTAC-3’ |
|  |  | R: 5’-TGTTGACAGTGGTATTTCTGGTG-3’ |
| *Rela* | 19697 | F: 5’-AGGCTTCTGGGCCTTATGTG-3’ |
|  |  | R: 5’-TGCTTCTCTCGCCAGGAATAC-3’ |
| *Actb* | 11461 | F: 5’-GGAAAAGAGCCTCAGGGCAT-3’ |
|  |  | R: 5’-GAAGAGCTATGAGCTGCCTGA-3’ |

1. **Western blot**

The lung/liver tissues were homogenized in RIPA lysis buffer (P0013B, Beyotime) and protease inhibitor (GRF101, Epizyme), and the supernatant was collected by centrifugation after sufficient lysis. The protein concentration was determined using the BCA method, and 5× loading buffer (P0015, Beyotime) was added to prepare samples with balanced concentration. Depending on the molecular weight of the target protein, a 6%-15% SDS-PAGE gel (P0012, Beyotime) was used and vertical electrophoresis was performed at a constant voltage of 80V for 1 to 2 hours. The proteins were transferred onto a 0.45 μm nitrocellulose membrane, which was blocked using protein-free rapid blocking buffer (PS108, Epizyme). Incubate blot in primary antibody (**Supplementary Table 4**) overnight at 4°C. Wash the membrane 4 times for 5 minutes each at room temperature in PBS + 0.1% Tween-20 with gentle shaking. Incubate blot in fluorescently-labeled secondary antibody (926-32211, LI-COR) for 1 hour at room temperature and avoid light exposure, and then wash the membrane again. Scan the membrane using Odyssey infrared imaging system (LI-COR, USA). Grayscale analysis was performed using the ImageJ software (National Institutes of Health, USA).

**Supplementary Table 4.** Antibodies used in the present study

| Antibody | Dilution | Manufacturer | Code |
| --- | --- | --- | --- |
| FGF21 | 1:1000 | Abcam | 171941 |
| FGF21* | 1:1000 | R&D system | AF3057 |
| NLRP3 | 1:1000 | Cell Signaling Technology | 15101 |
| ASC | 1:1000 | Cell Signaling Technology | 67824 |
| HMGB1 | 1:1000 | Cell Signaling Technology | 6893 |
| Caspase1 | 1:1000 | Cell Signaling Technology | 24232 |
| Cleaved Caspase1 | 1:1000 | Cell Signaling Technology | 89332 |
| Cleaved IL-1β | 1:1000 | Cell Signaling Technology | 63124 |
| IL-18 | 1:1000 | Cell Signaling Technology | 57058 |
| GSDMD | 1:1000 | Cell Signaling Technology | 46451 |
| β-actin | 1:1000 | Beyotime | AF5001 |

*This antibody was used in the additional experiment during revision.

**Supplementary figures**


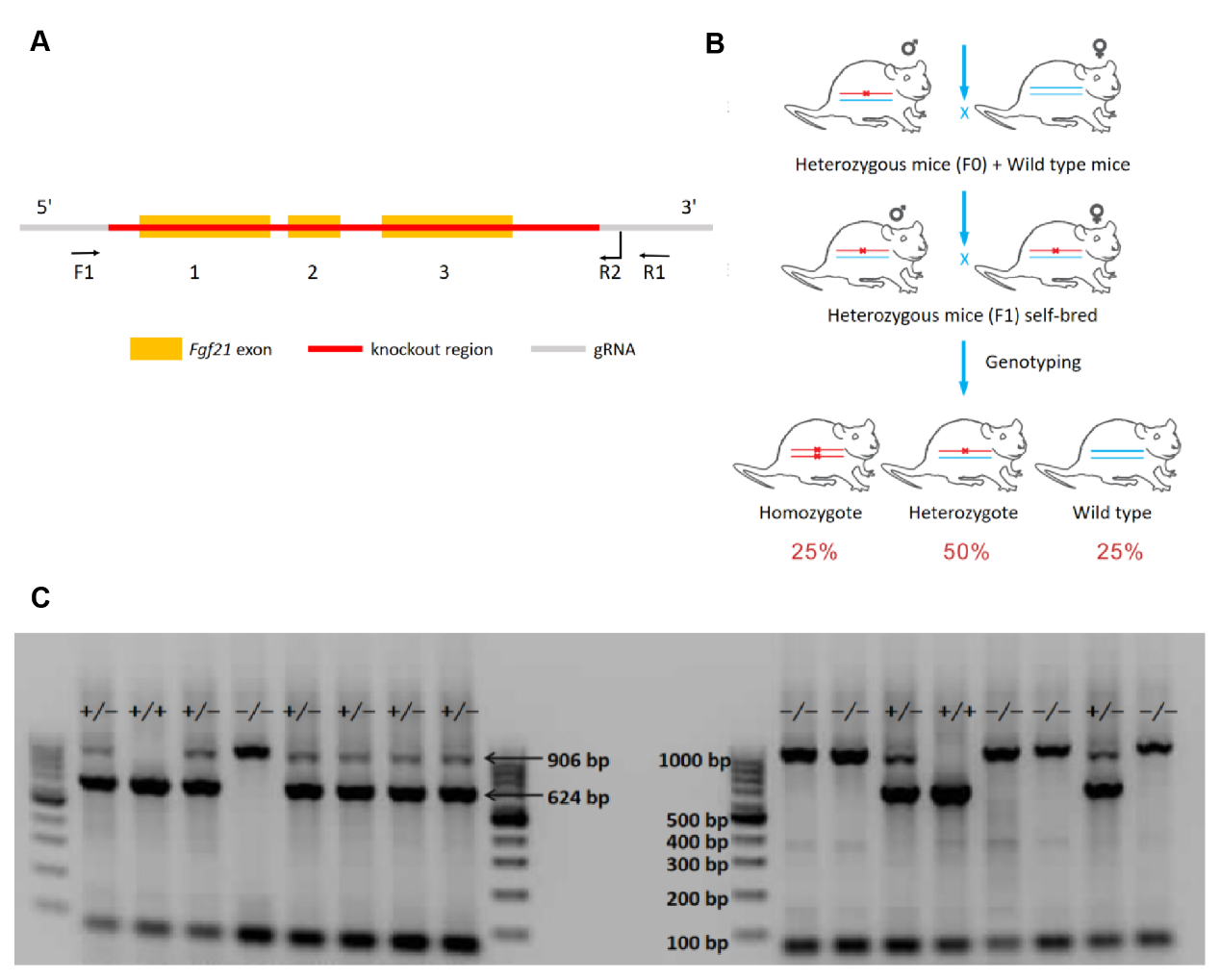


**Supplementary Figure 1. FGF21 knockout mouse.**

**(A)** The knockout strategy; **(B)** The breeding route; **(C)** The exposure image of agarose gel, one band at 906 bp refers to the homozygote, one band at 624 bp refers to the wild type, and two bands at 624/906 bp refer to heterozygote.


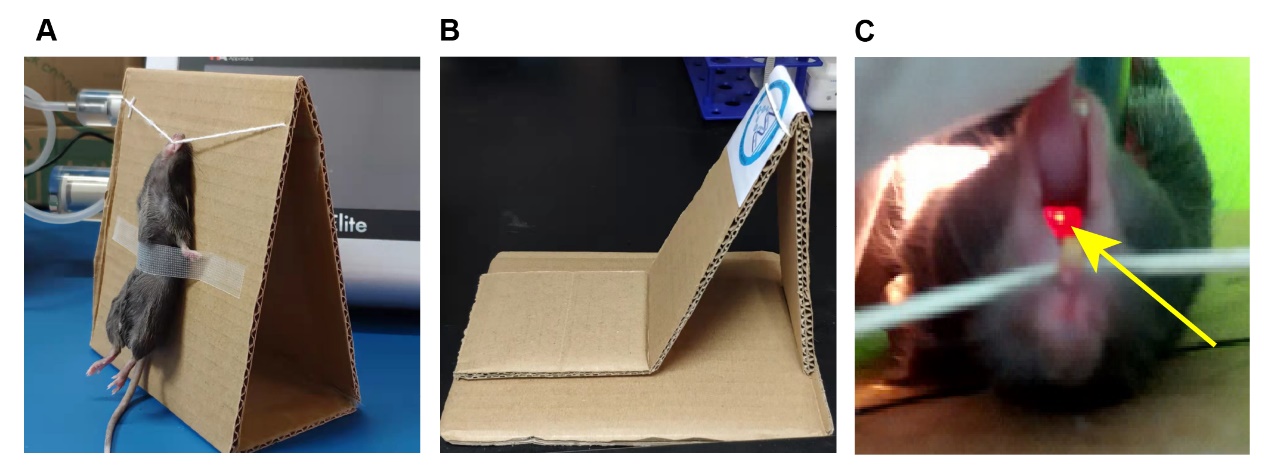


**Supplementary Figure 2. Transoral intubation in mouse VILI model.**

**(A).** The position of the mouse during intubation; **(B).** The modified device used in intubation; **(C).** The glottis of the mouse (yellow arrow).


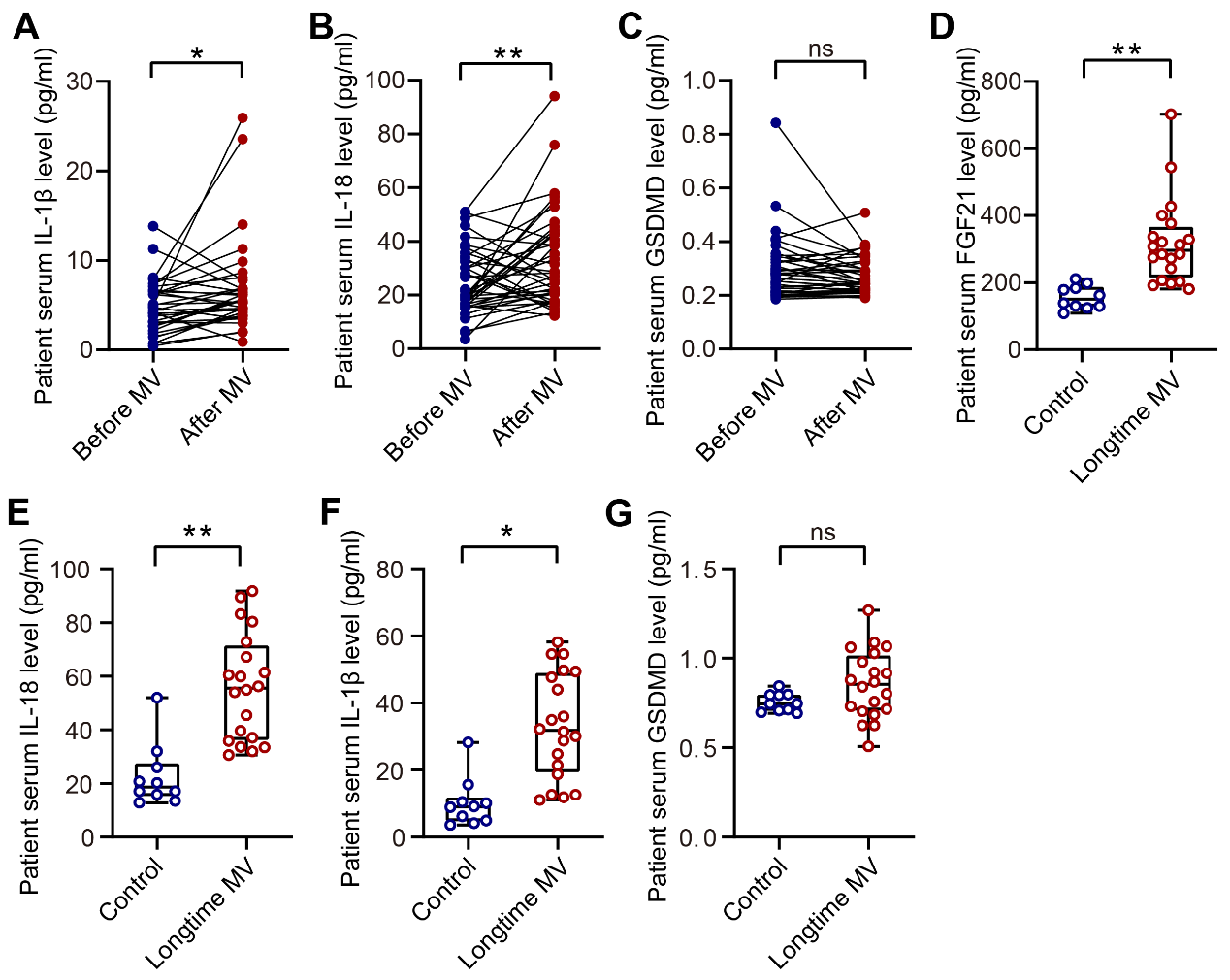


**Supplementary Figure 3. Measurement of FGF21, inflammatory cytokines and pyroptosis marker in patients.**

**(A).** Serum IL-1β levels before/after mechanical ventilation (*N*=40); **(B).** Serum IL-18 levels before/after mechanical ventilation (*N*=40); **(C).** Serum GSDMD levels before/after mechanical ventilation (*N*=40); **(D).** Serum FGF21 levels in patients with long-term mechanical ventilation (*N*=20) and healthy volunteers (*N*=10); **(E).** Serum IL-1β levels in patients with long-term mechanical ventilation (*N*=20) and healthy volunteers (*N*=10); **(F).** Serum IL-18 levels in patients with long-term mechanical ventilation (*N*=20) and healthy volunteers (*N*=10); **(G).** Serum GSDMD levels in patients with long-term mechanical ventilation (*N*=20) and healthy volunteers (*N*=10). **P*<0.05, ***P*<0.01, ns, no significance.


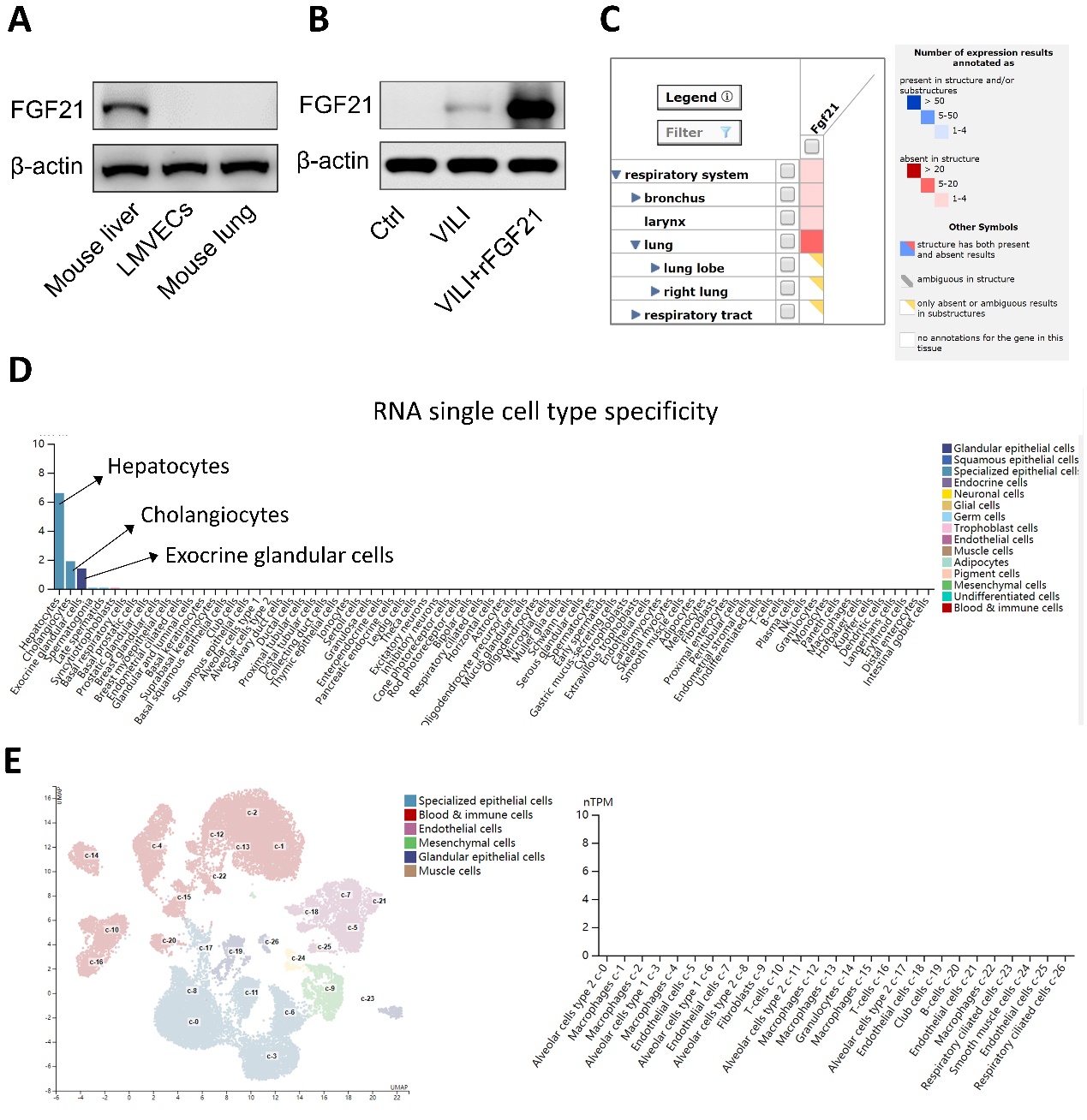


**Supplementary Figure 4. FGF21 expression in mouse, primary cells, and human.**

**(A)** Western blotting shows that FGF21 was expressed in mouse liver, and was not detectable in primary lung microvascular endothelial cells and total lung tissues; **(B)** Western blotting using mouse lung tissues shows that FGF21 was not expressed in lungs of control mice, but was expressed in lungs after VILI modeling, and further increased after rFGF21 treatment. **(C)** Refine results from the Mouse Genome Informatics database show that Fgf21 gene was absent or ambiguous in mouse lung tissues. Source: [www.informatics.jax.org/marker/MGI:1861377](http://www.informatics.jax.org/marker/MGI:1861377); **(D)** Overview of human single cell types shows that FGF21 is enhanced in hepatocytes, cholangiocytes, and exocrine glandular cells. Source: [www.proteinatlas.org/ENSG00000105550-FGF21/single+cell+type](http://www.proteinatlas.org/ENSG00000105550-FGF21/single+cell+type); **(E)** Single cell sequencing shows that FGF21 expression is not detected in the lung. Source: <https://www.proteinatlas.org/ENSG00000105550-FGF21/single+cell+type/lung>.


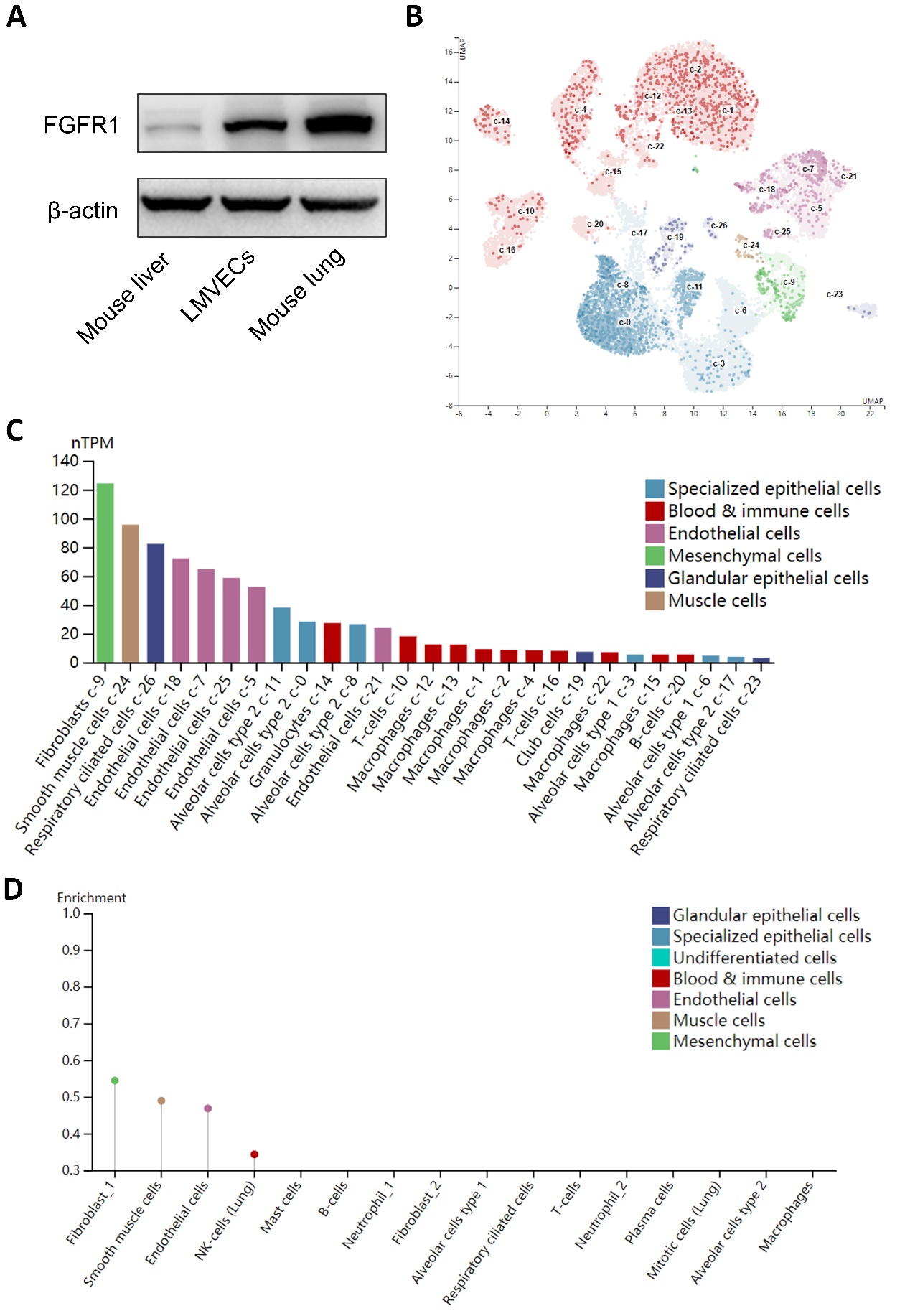


**Supplementary Figure 5. FGFR1 expression in mouse and human.**

**(A)** Western blotting shows that FGFR1 is expressed at a low level in mouse liver tissues, while it was expressed at a higher level in both primary lung microvascular endothelial cells and total lung tissues. **(B&C)** Human single cell sequencing indicates that FGFR1 is highly expressed in fibroblasts, smooth muscle cells, respiratory ciliated cells, and endothelial cells, and is relatively low in type I/II alveolar cells. Source: www.proteinatlas.org/ENSG00000077782-FGFR1/single+cell+type/lung; **(D)** Cell type enrichment analysis indicates that FGFR1 is enriched in fibroblasts, smooth muscle cells and endothelial cells but not type I/II alveolar cells. Source: [www.proteinatlas.org/ENSG00000077782-FGFR1/tissue+cell+type/lung](http://www.proteinatlas.org/ENSG00000077782-FGFR1/tissue+cell+type/lung).


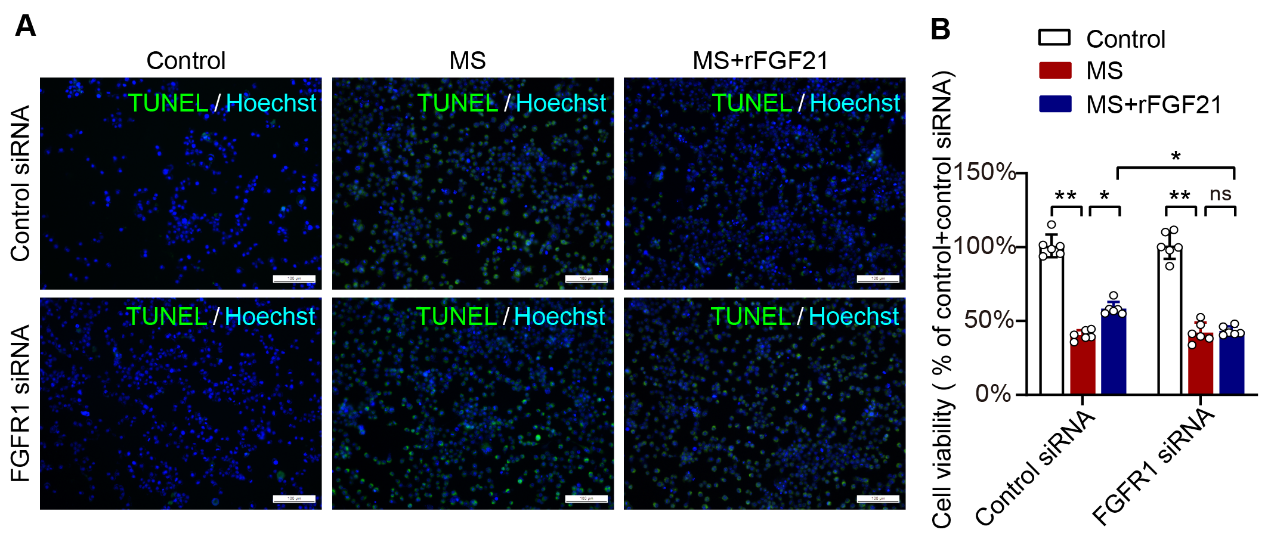


**Supplementary Figure 6. FGFR1 mediates the protective effect of FGF21 on lung microvascular endothelial cells.**

1. Lung microvascular endothelial cells were transfected with FGFR1 siRNA or control siRNA. After 72 hours, the culture medium was changed, and the cells were subjected to mechanical stretch for 4 hours with or without rFGF21 treatment (100 nM). The nucleus was stained blue and the dead cells were stained green. Scale bar=100 μm. **(B)** Cell viability was assessed by CCK8 method. *N*=6, **P*<0.05, ***P*<0.01, ns, no significance.
